# Supplementary material for: An outer membrane porin-lipoprotein complex modulates elongasome movement to establish cell curvature in Rhodospirillum rubrum
Source: Nat Commun. 2024 Sep 2;15:7616. doi: 10.1038/s41467-024-51790-z (PMC11369160; doi:10.1038/s41467-024-51790-z)
Supplement: Supplementary file 1 — Supplementary Information [file 41467_2024_51790_MOESM1_ESM.pdf]

Supplementary information

An outer membrane porin-lipoprotein complex modulates elongasome movement to establish cell curvature in *Rhodospirillum rubrum*

Sebastian Pöhl, Giacomo Giacomelli, Fabian M. Meyer, Volker Kleeberg, Eli J. Cohen, Jacob Biboy, Julia Rosum, Timo Glatter, Waldemar Vollmer, Muriel C. F. van Teeseling, Johann Heider, Marc Bramkamp, Martin Thanbichler

Supplementary figures

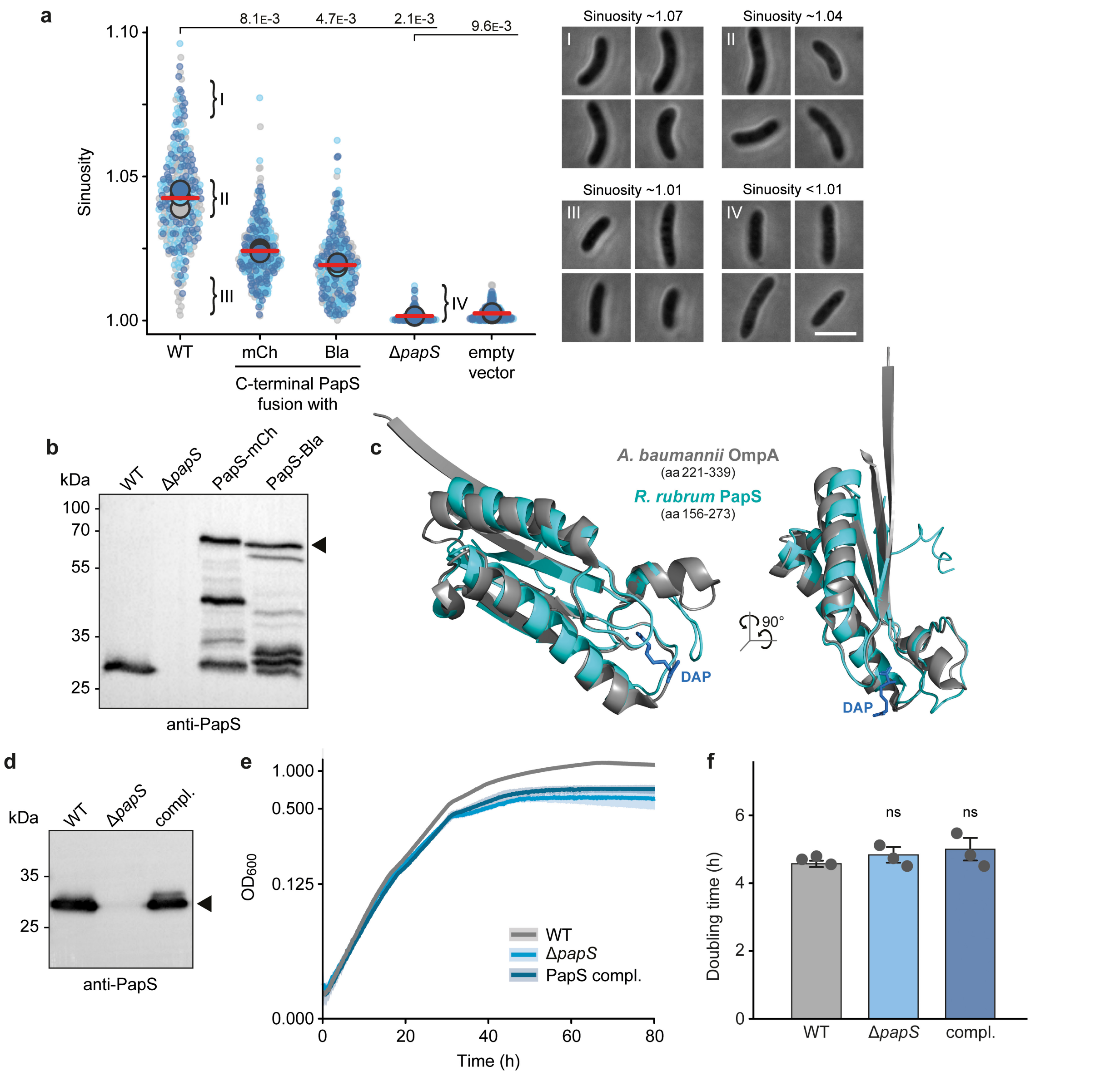

Supplementary Figure 1. ***In vivo*** **characterization of the properties of PapS. (a)** Superplots showing the distribution of cell sinuosities for cells producing PapS-mCh (SP01) or PapS-Bla (SP231) fusion proteins in place of the respective native proteins or harboring the empty vector pRXMCS-2 (SP23). The sinuosity distributions of wild-type (S1) and Δ*papS* (JR52) cells from **Figure 1f** are shown as a reference. Small dots represent the data obtained in three independent biological replicates (shown in dark blue, light blue and grey; n=100 cells per replicate). Large dots represent the mean value of the three datasets. The red horizontal line indicates the average of these three mean values. The statistical significance (*p* value) of differences between strains is indicated (unpaired two-sided Welch's t-test). The images on the right show representative cells with sinuosities that fall into the four ranges indicated by Roman numerals. Bar: 3 µm. **(b)** Immunoblot analysis of strains producing PapS-mCh (SP01) and PapS-Bla (SP231) in place of the native PapS protein. Wild-type (S1) and Δ*papS* (JR52) cells were analyzed as controls. Proteins were detected with an anti-PapS antibody. The predicted molecular weights of the processed proteins are 27.0 kDa (PapS), 55.4 kDa (PapS-mCh) and 57.4 kDa (PapS-Bla). The bands of lower molecular weigth represent degradation products. **(c)** Superimposition of the crystal structure of the OmpA-like domain of *A. baumannii* OmpA in complex with diaminopimelic acid (DAP) (PDB accession code: 3TD4) [1] and the AlphaFold2 [2] model of the OmpA domain of *R. rubrum* PapS. **(d)** Immunoblot analysis of wild-type (S1) and Δ*papS* (JR52) cells and of Δ*papS* cells producing *papS* from a low-copy number plasmid (compl.). Proteins were detected with an anti-PapS antibody. The predicted molecular weight of PapS (indicated by an arrowhead) is 27.0 kDa. **(e)** Growth curves of the strains analyzed in panel c. Lines represent the mean of 3 biological replicates. The standard deviations are indicated by the shaded areas. **(f)** Doubling times of the indicated strains in the exponential growth phase, as derived from the growth curves shown in panel d.

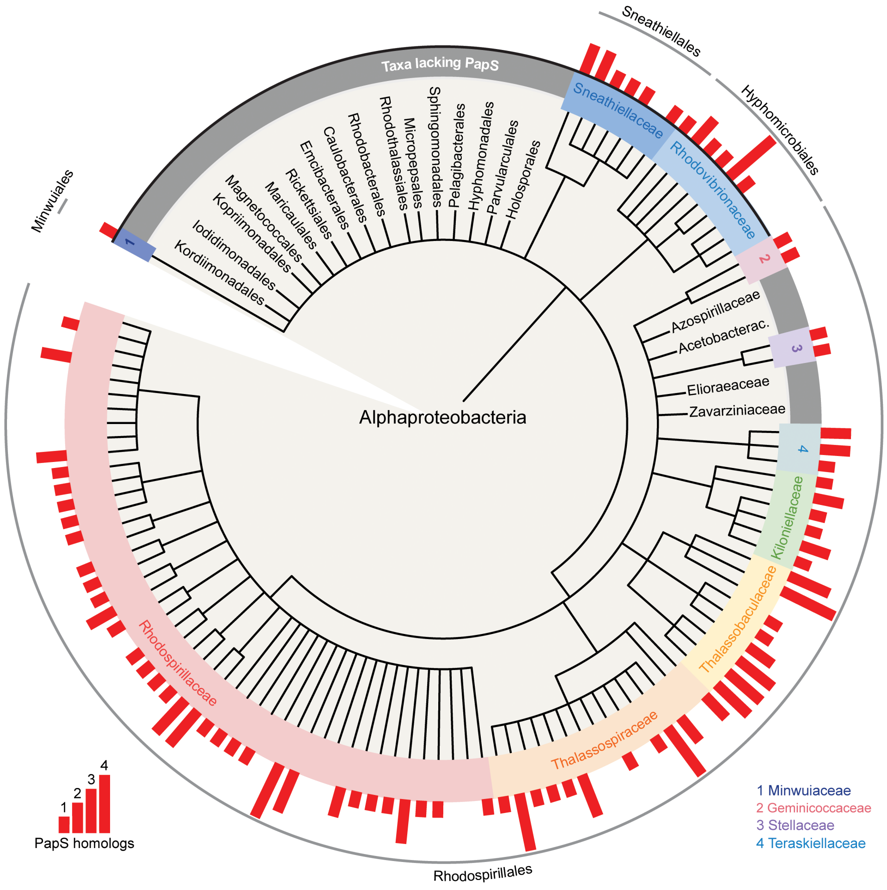

Supplementary Figure 2. Conservation of PapS in the *Rhodospirillales*. The BlastP webserver was used to identified genomes that contained one or multiple PapS-encoding genes, as defined by hits with an e-value of 10^{-15} or lower). In cases where PapS homologs were found in multiple strains per species, only one representative strain was chosen for further analysis. Subsequently, a phylogenetic tree of the final species set was generated with phyloT and visualized using the iTOL server. For each species shown, the number of PapS homologs encoded in the genome is indicated by a red bar.

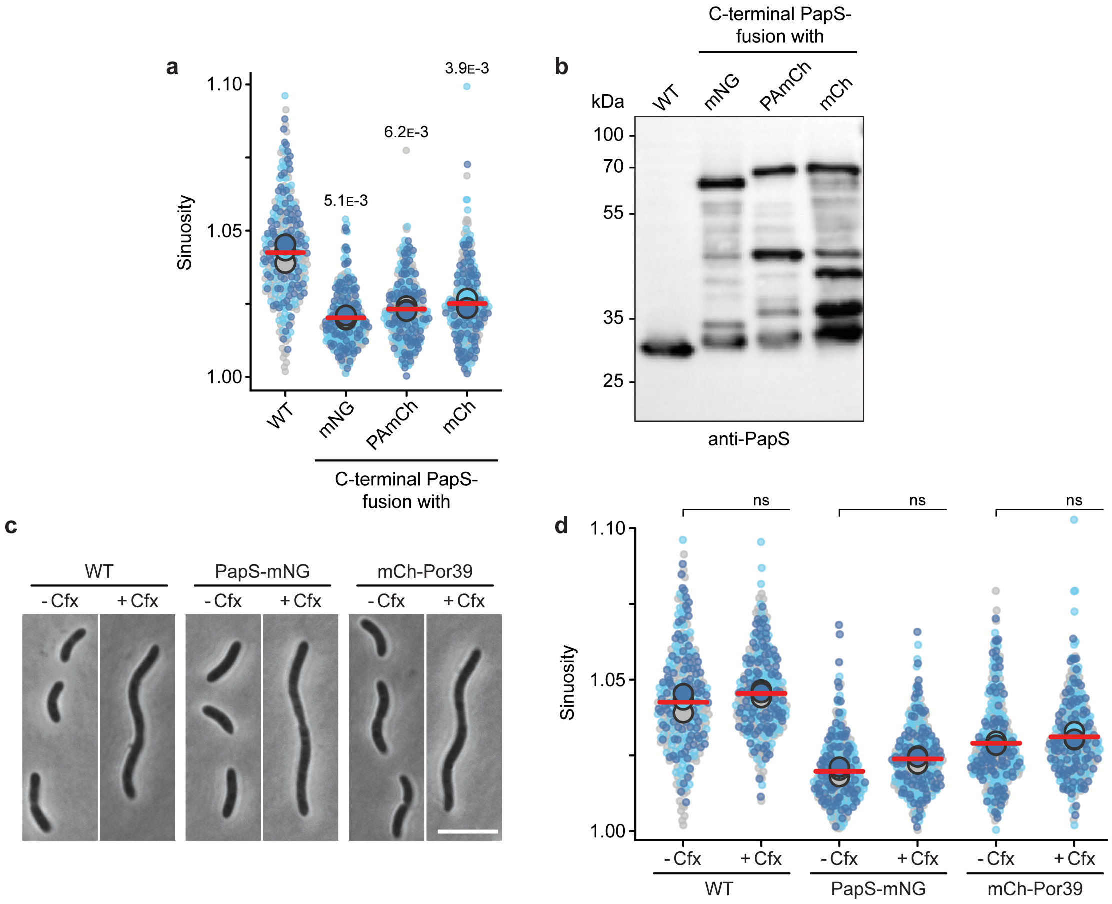

Supplementary Figure 3. Analysis of PapS localization and function. (a) Superplots showing the distribution of cell sinuosities in populations of cells producing PapS-mNG (SP02) or PapS-PAmCh (SP04) in place of the native PapS protein or of Δ*papS* cells producing PapS-mCh from a low-copy number plasmid (JR56). The sinuosity distributions of wild-type cells from **Figure 1f** are shown as a reference. The data are presented as described in **Supplementary Figure 1a** (n=100 cells per replicate). The statistical significance (*p* value) of differences between strains is indicated (unpaired two-sided Welch's t-test). **(b)** Immunoblot analysis of the strains analyzed in panel a. Proteins were detected with an anti-PapS antibody. The predicted molecular weights of the processed proteins are 27.0 kDa (PapS), 55.3 kDa (PapS-mNG), 55.5 kDa (PapS-PAmCh) and 55.4 kDa (plasmid-encoded PapS-mCh). **(c)** Phase-contrast micrographs showing representative *R. rubrum* wild-type (S1) cells and cells producing PapS-mNG (SP02) or mCh-Por39 (SP222) after cultivation in the absence (- Cfx) or presence (+ Cfx) of cefalexin (n=3 biological replicates). Bar: 5 µm. **(d)** Superplots showing the distribution of cell sinuosities in the cell populations described in panel c. Data are presented as described in **Supplementary Figure 1a** (n=100 cells per replicate). The statistical significance (*p* value) of differences between strains is indicated (unpaired two-sided Welch's t-test; ns, not significant).

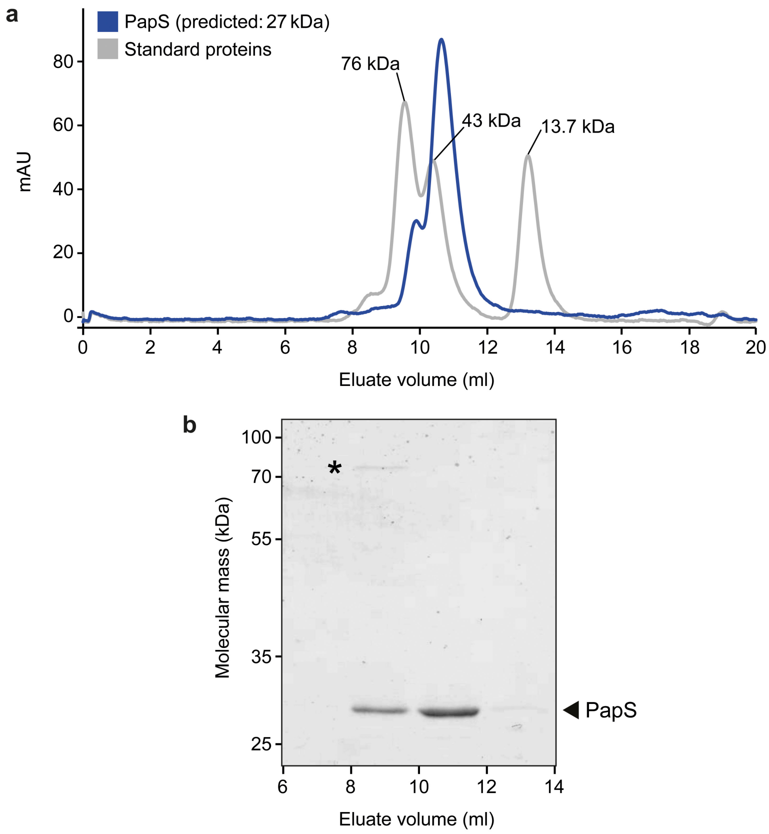

Supplementary Figure 4. Determination of the oligomerization state of PapS. (a) Size-exclusion chromatography of purified PapS. A mixture of Ribonuclease A (13.7 kDa), Ovalbumin (43 kDa) and Conalbumin (76 kDa) was analyzed as a reference. **(b)** SDS gel showing the protein content of the four eluate fractions comprising the major peak (6-14 ml) in the experiment shown in panel a. The predicted molecular weight of PapS is 27.0 kDa (indicated by an arrowhead). The asterisk indicates a contaminating protein likely responsible for the small shoulder in the PapS peak.

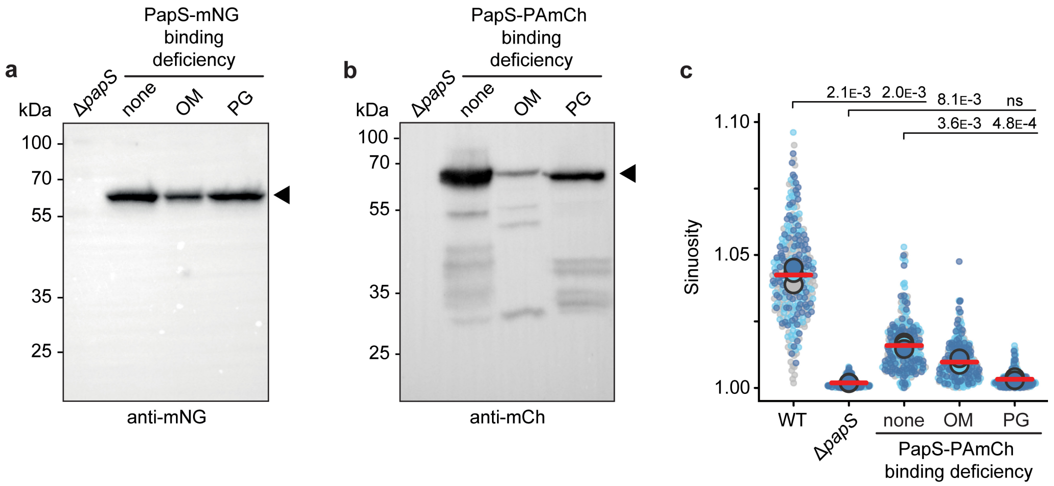

Supplementary Figure 5. Analysis of strains producing fluorescently tagged PapS variants deficient in outer-membrane or peptidoglycan binding. (a) Immunoblot analysis of *R. rubrum* Δ*papS* cells producing PapS-mNG (SP136; none), a PapS-mNG variant lacking the outer-membrane (OM) lipid anchor (SP184) or a PapS-mNG variant defective in peptidoglycan (PG) binding (SP185) from a low-copy number plasmid. Cells of the parential Δ*papS* strain (JR52) were analyzed as a negative control. Proteins were detected with an anti-mNeonGreen antibody. The predicted molecular weight of PapS-mNG (indicated by an arrowhead) is 55.3 kDa. **(b)** Immunoblot analysis of *R. rubrum* Δ*papS* cells producing PapS-PAmCh (SP165), a PapS-PAmCh variant lacking the outer-membrane lipid anchor (SP166) or a PapS-PAmCh variant defective in peptidoglycan binding (SP167) from a low-copy number plasmid. The Δ*papS* mutant (JR52) was analyzed as a negative control. Proteins were detected with an anti-mCherry antibody. The predicted molecular weight of PapS-PAmCh (indicated by an arrowhead) is 55.5 kDa. **(c)** Superplots showing the distribution of cell sinuosities in populations of the strains analyzed in panel b. The sinuosity distributions of wild-type (S1) and Δ*papS* (JR52) cells from **Figure 1f** are shown as a reference. The data are presented as described in **Supplementary Figure 1a** (n=100 cells per replicate). The statistical significance (*p* value) of differences between strains is indicated (unpaired two-sided Welch's t-test; ns, not significant).

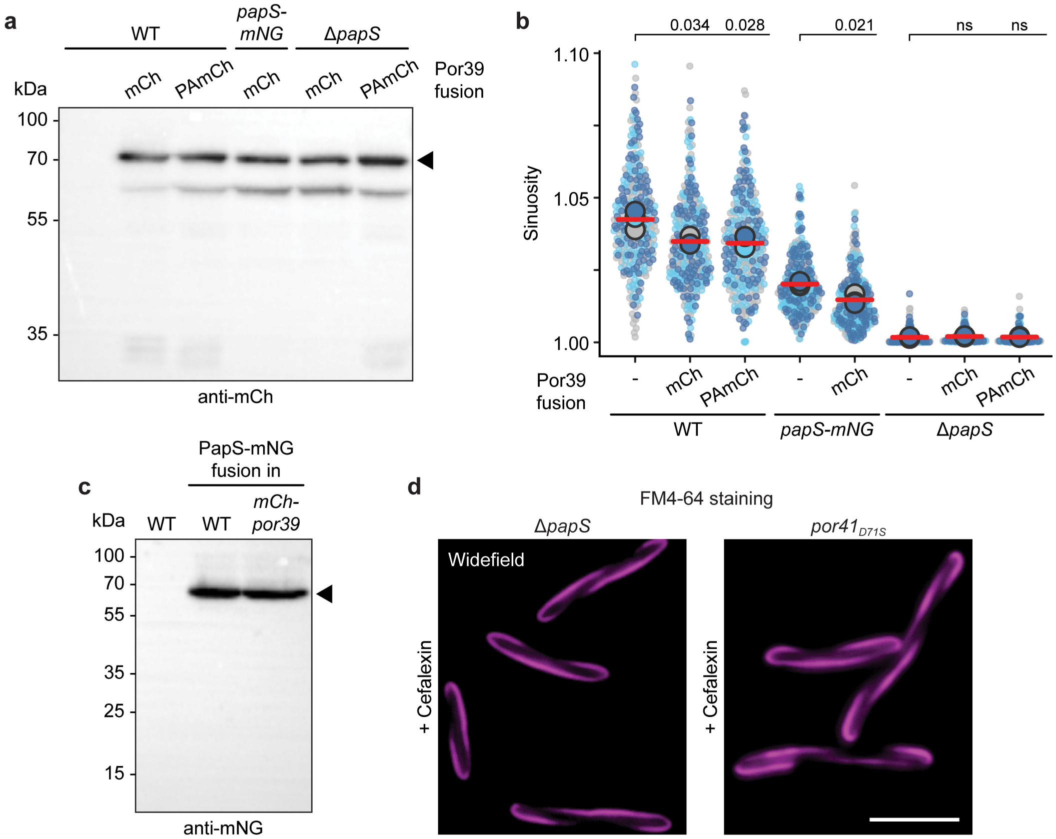

Supplementary Figure 6. **Analysis of strains producing fluorescently tagged Por39 variants. (a)** Immunoblot analysis of cells producing mCh-Por39 (SP222) or PAmCh-Por39 (SP173) in the wild-type background, mCh-Por39 in the *papS-mNG* background (SP06) or mCh-Por39 (SP12) or PAmCh-Por39 (SP177) in the Δ*papS* background. Wild-type (S1) cells were analyzed as a negative control. Proteins were detected with an anti-mCherry antibody. The predicted molecular weights of the of the processed fusion proteins (indicated by an arrowhead) are 63.0 kDa (mCh-Por39) and 61.3 kDa (PAmCh-Por39). **(b)** Superplots showing the distribution of cell sinuosities in populations of the strains described in panel a. The sinuosity distributions of wild-type (S1) and Δ*papS* (JR52) cells from **Figure 1f** are shown as a reference. The data are presented as described in **Supplementary Figure 1a** (n=100 cells per replicate). The statistical significance (*p* value) of differences between strains is indicated (unpaired two-sided Welch's t-test; ns, not significant). **(c)** Immunoblot analysis of cells producing PapS-mNG in place of the endogenous PapS protein in an otherwise wild-type (SP04) or *mCh-por39* (SP06) background. Proteins were detected with an anti-mNeonGreen antibody. The predicted molecular weight of processed PapS-mNG is 55.3 kDa. **(d)** FM4-64 staining of *R. rubrum* Δ*papS* (JR52) and *por41*$_{D71S}$ (SP150) cells. Shown are representative cells (n=3 biological replicates), analyzed by widefield microscopy. Bar: 5 µm.

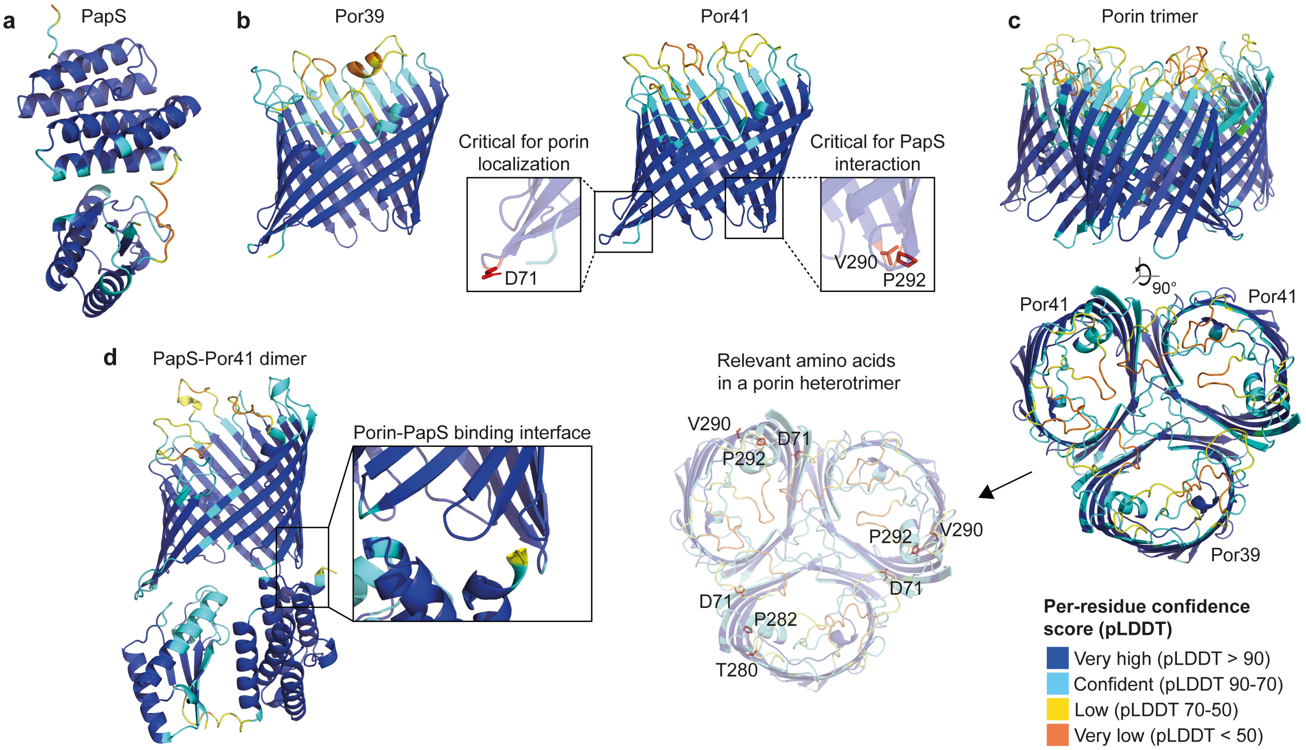

Supplementary Figure 7. Structural models of the proteins involved in the PapS systems. (a-d) Predicted molecular structures of PapS, Por39 and Por41, a Por41$_2$-Por39 trimer and a PapS-Por41 dimer, generated with AlphaFold2 [2] or AlphaFold-Multimer [3], respectively. The coloring of the structures indicates the per-residue confidence score (pLDDT). Amino acids in the two porins that have been exchanged in this study are highlighted in the corresponding structures. The structural coordinates and error estimates for the different models are provided in **Supplementary Data 7**.

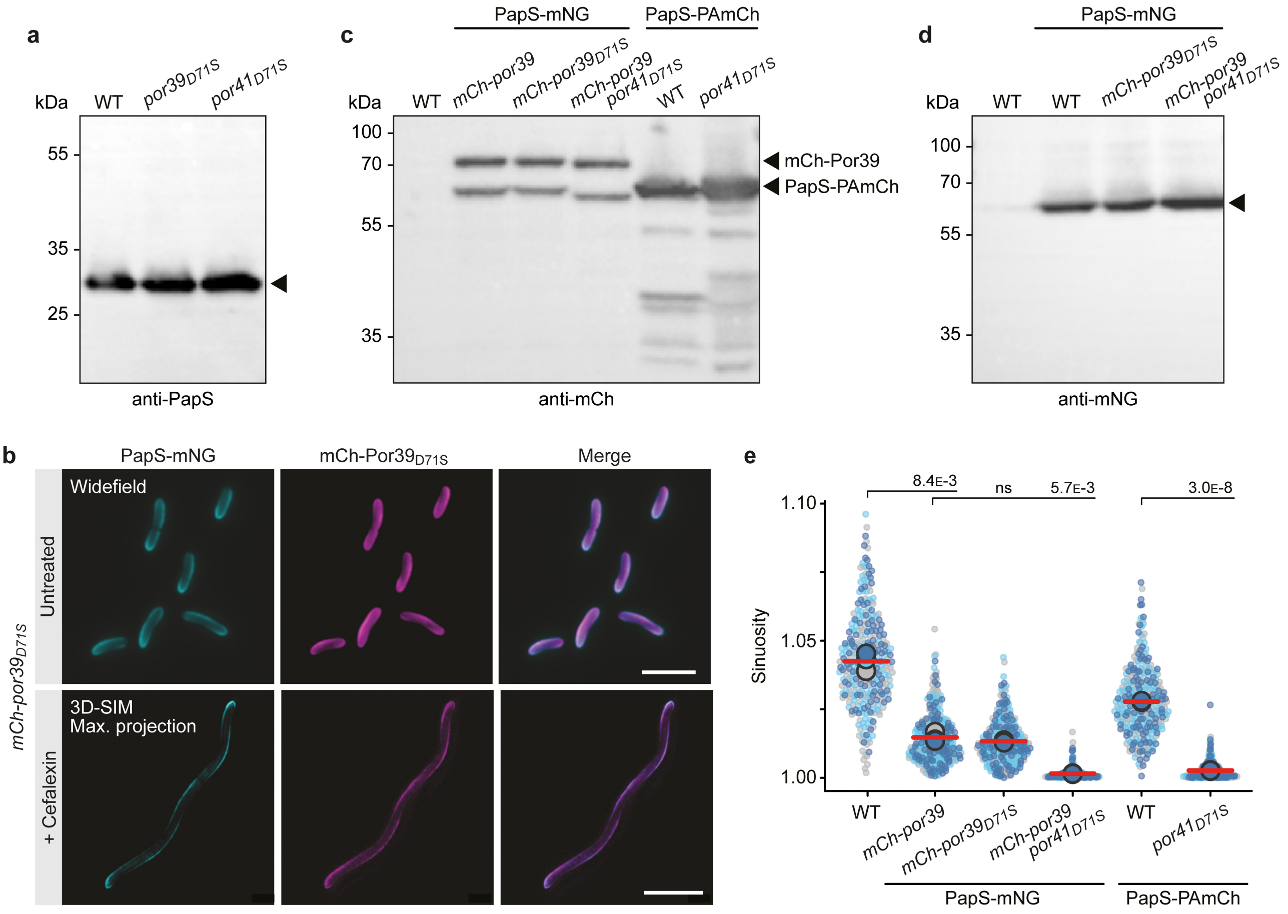

Supplementary Figure 8. Analysis of Por39 and Por41 variants carrying the D71S exchange. (a) Immunoblot analysis detecting PapS in wild-type (S1), *por39*$_{D71S}$ (SP215) and *por41*$_{D71S}$ (SP150) cells. Proteins were detected with an anti-PapS antibody. The predicted molecular weight of processed PapS is 27.0 kDa (indicated by an arrowhead). **(b)** Colocalization of PapS-mNG and mCh-Por39$_{D71S}$ in cells (SP130) cultivated in the absence (untreated) or presence (+) of cefalexin, as analyzed by widefield epifluorescence microscopy or 3D-SIM, respectively. Bar: 5 µm. The area overlap of the PapS-mNG and mCh-Por39$_{D71S}$ signals in the 3D-SIM images is 80.4% (see also Supplementary Data 3). **(c)** Immunoblot analysis of cells producing PapS-mNG in the *mCh-por39* (SP06), *mCh-por39*$_{D71S}$ (SP130) or *mCh-por39 por41*$_{D71S}$ (SP131) background or producing PapS-PAmCh in the wild-type (SP04) or *por41*$_{D71S}$ (SP183) background. Wild-type (S1) cells were analyzed as a negative control. Proteins were detected with an anti-mCherry antibody. The predicted molecular weights of the processed proteins (indicated by arrowheads) are 63.0 kDa (mCh-Por39) and 55.5 kDa (PapS-PAmCh). **(d)** Immunoblot analysis of cells producing PapS-mNG in the wild-type (SP02), *mCh-pro39*$_{D71S}$ (SP130) or *mCh-por39 mCh-por41*$_{D71S}$ (SP131) background. Proteins were detected with an anti-mNeonGreen antibody. Wild-type (S1) cells were analyzed as a negative control. The predicted molecular weight of processed PapS-mNG is 55.3 kDa (indicated by an arrowhead). **(e)** Superplots showing the distribution of cell sinuosities in populations of the strains analyzed in panel c. The sinuosity distributions of wild-type (S1) cells from Figure 1f are shown as a reference. The data are presented as described in Supplementary Figure 1a (n=100 cells per replicate). The statistical significance (*p* value) of differences between strains is indicated (unpaired two-sided Welch's t-test; ns, not significant).

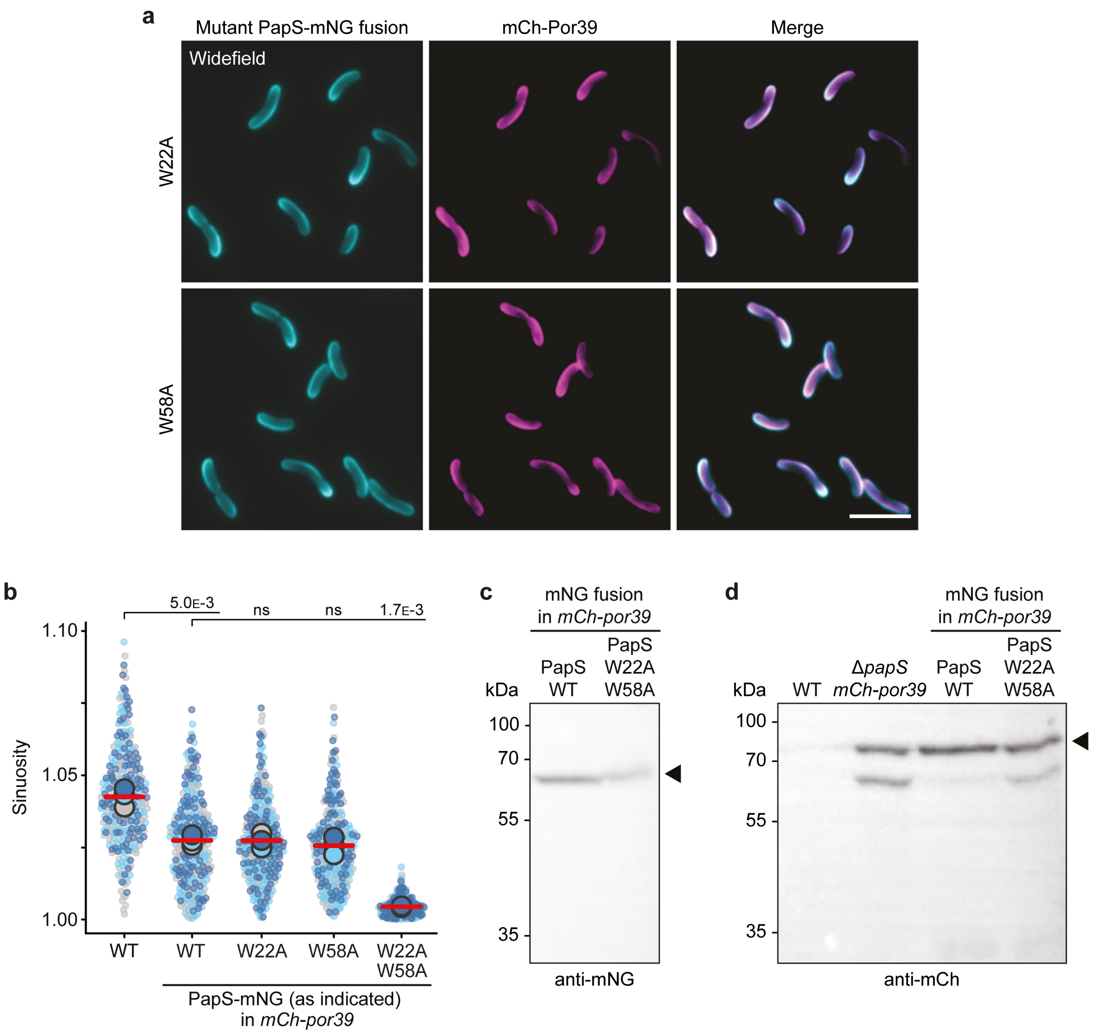

Supplementary Figure 9. Analysis of PapS-mNG variants with exchanges in the predicted porin-binding site. (a) Colocalization of $\text{PapS}_{\text{W22A}}$-mNG (SP143) or $\text{PapS}_{\text{W58A}}$-mNG (SP144) with mCh-Por39 by widefield epifluorescence microscopy. Bar: 5 µm. **(b)** Superplots showing the distribution of cell sinuosities in populations of Δ*papS* cells producing PapS-mNG (SP126), $\text{PapS}_{\text{W22A}}$-mNG (SP143), $\text{PapS}_{\text{W58A}}$-mNG (SP144) or $\text{PapS}_{\text{W22A/W58A}}$-mNG (SP146) from a low-copy number plasmid in an *mCh-por39* background. The sinuosity distribution of wild-type (S1) cells from **Figure 1f** are shown as a reference. The data are presented as described in **Supplementary Figure 1a** (n=100 cells per replicate). The statistical significance (*p* value) of differences between strains is indicated (unpaired two-sided Welch's t-test; ns, not significant). **(c)** Immunoblot analysis of Δ*papS* cells producing PapS-mNG (SP126) or $\text{PapS}_{\text{W22A/W58A}}$-mNG (SP146) from a low-copy number plasmid in an *mCh-por39* background. Proteins were detected with an anti-mNeonGreen antibody. The predicted molecular weight of the processed PapS-mNG variants is 55.4 kDa (indicated by an arrowhead). **(d)** Immunoblot analysis of the strains described in panel c, performed using an anti-mCherry antibody. The *R. rubrum* wild type (S1) and the parential Δ*papS mCh-por39* strain (SP12) were analyzed as controls. The predicted molecular weight of processed mCh-Por39 is 63.0 kDa (indicated by an arrowhead).

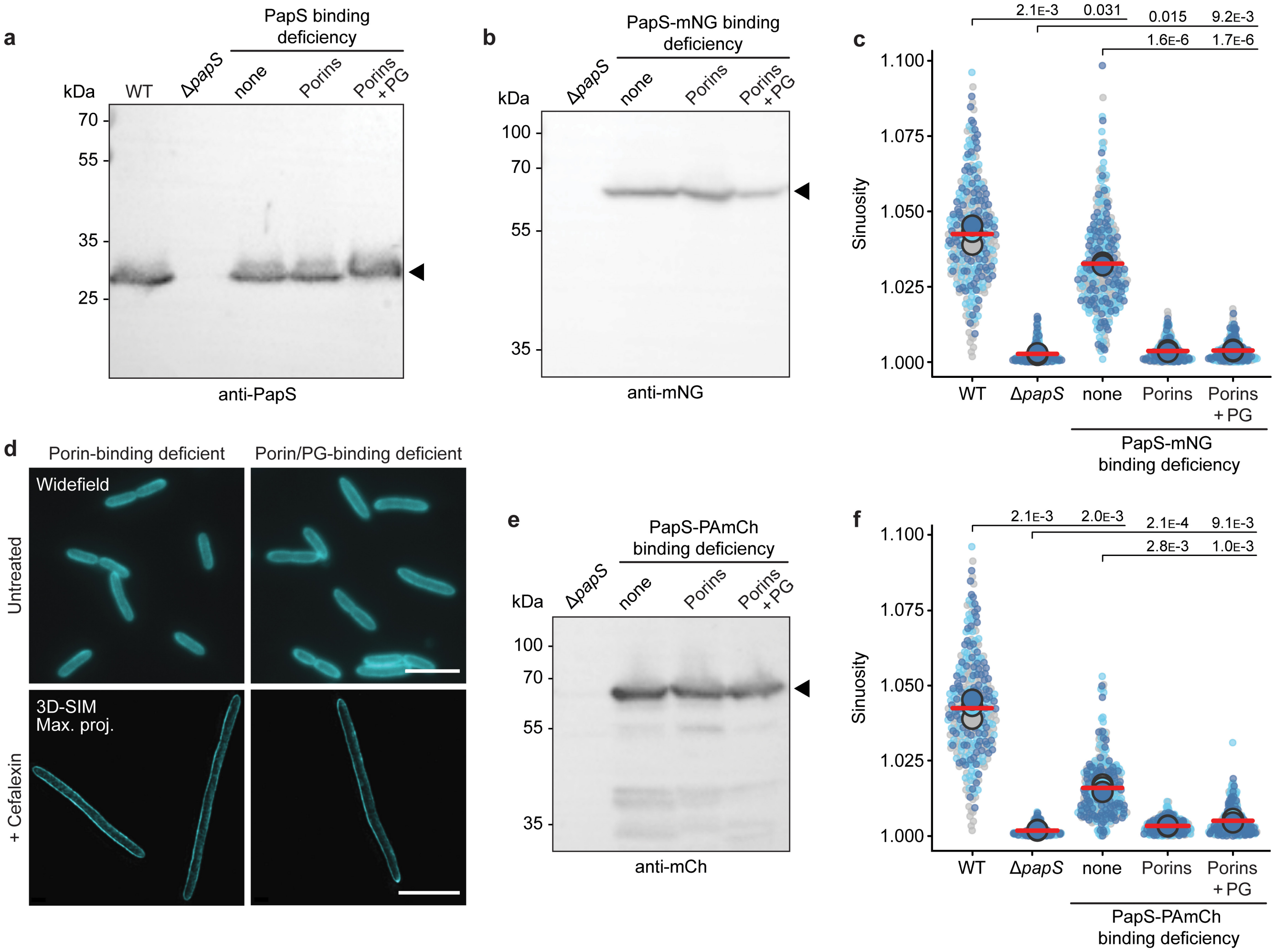

Supplementary Figure 10. Analysis of strains producing fluorescently tagged PapS variants defective in porin or peptidoglycan binding. **(a)** Immunoblot analysis of Δ*papS* cells producing wild-type PapS (none; JR54) or mutant variants thereof defective in porin-binding (SP186) or both porin and peptidoglycan binding (SP187) from a low-copy number plasmid. Wild-type (S1) and Δ*papS* (JR52) cells were analyzed as controls. Proteins were detected with an anti-PapS antibody. The predicted molecular weight of processed PapS is 27.0 kDa (indicated by an arrowhead). **(b)** Immunoblot analysis of Δ*papS* cells producing PapS-mNG (none; SP136) or mutant variants thereof defective in porin-binding (SP197) or both porin and peptidoglycan binding (SP174) from a low-copy number plasmid. The Δ*papS* mutant (JR52) was analyzed as a negative control. Proteins were detected with an anti-mNeonGreen antibody. The predicted molecular weight of processed PapS-mNG is 55.3 kDa (indicated by an arrowhead). **(c)** Superplots showing the distribution of cell sinuosities in populations of the *R. rubrum* strains described in panel b. The sinuosity distributions of wild-type (S1) and Δ*papS* (JR52) cells from Figure 1f and of Δ*papS* cells producing PapS-mNG (SP136) from Figure 3c were replotted as a reference. The data are presented as described in Supplementary Figure 1a (n=100 cells per replicate). The statistical significance (*p* value) of differences between strains is indicated (unpaired two-sided Welch's t-test). **(d)** Localization patterns of PapS-mNG variants defective in porin binding (SP197) or both porin and peptidoglycan binding (SP174) in cells grown in the absence (untreated) or presence (+) of cefalexin, as visualized by widefield epifluorescence or 3D-SIM, respectively. Bar: 5 µm. **(e)** Immunoblot analysis of Δ*papS* cells producing PapS-PAmCh (none; SP165) or mutant variants thereof defective in porin-binding (SP168) or both porin and peptidoglycan binding (SP175) from a low-copy number plasmid. The Δ*papS* mutant (JR52) was analyzed as a negative control. Proteins were detected with an anti-mCherry antibody. The predicted molecular weight of processed PapS-PAmCh is 55.5 kDa (indicated by an arrowhead). **(f)** Superplots showing the distribution of cell sinuosities in populations of the *R. rubrum* stains described in panel e. The results for wild-type (S1) and Δ*papS* (JR52) cells from Figure 1f and for Δ*papS* cells producing PapS-PAmCh (SP165) from Supplementary Figure 5d were replotted as a reference. The data are presented as described in Supplementary Figure 1a (n=100 cells per replicate). The statistical significance (*p* value) of differences between strains is indicated (unpaired two-sided Welch's t-test).

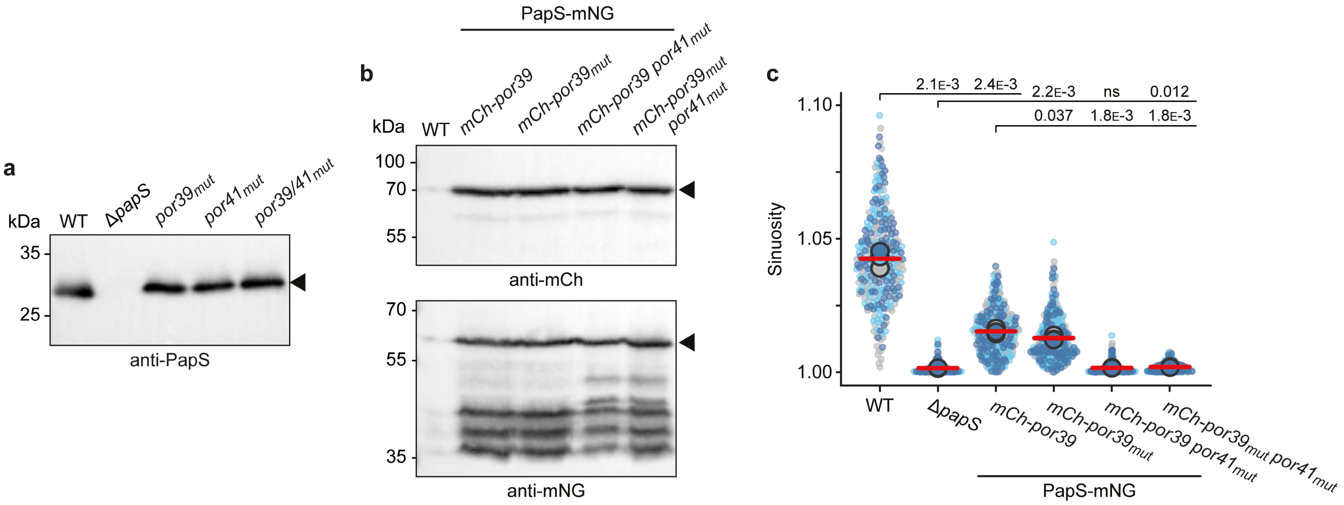

Supplementary Figure 11. Analysis of strains producing porin variants with substitutions in the predicted PapS binding site. (a) Immunoblot analysis of wild-type (S1), Δ*papS* (JR52), *por39*$_{mut}$ (SP235), *por41*$_{mut}$ (SP234) and *por39*$_{mut}$ *por41*$_{mut}$ (SP242) cells. Proteins were detected with an anti-PapS antibody. The predicted molecular weight of processed PapS is 27.0 kDa (indicated by an arrowhead). **(b)** Immunoblot analysis of cells producing PapS-mNG in place of the native PapS protein in the *mCh-por39* (SP06), *mCh-por39*$_{mut}$ (LMS23), *mCh-por39 por41*$_{mut}$ (LMS24) or *mCh-por39*$_{mut}$ *por41*$_{mut}$ (SP243) background. Wild-type (S1) cells were analyzed as a negative control. Proteins were detected with an anti-mCherry or an anti-mNeonGreen antibody. The predicted molecular weights of the processed proteins (indicated by arrowheads) are 63.0 kDa (mCh-Por39) and 55.4 kDa (PapS-mNG). **(c)** Superplots showing the distribution of cell sinuosities in populations of the *R. rubrum* stains described in panel b. The results for wild-type (S1) and Δ*papS* cells (JR52) were replotted from **Figure 1f** as a reference. The data are presented as described in **Supplementary Figure 1a** (n=100 cells per replicate). The statistical significance (*p* value) of differences between strains is indicated (unpaired two-sided Welch's t-test; ns, not significant).

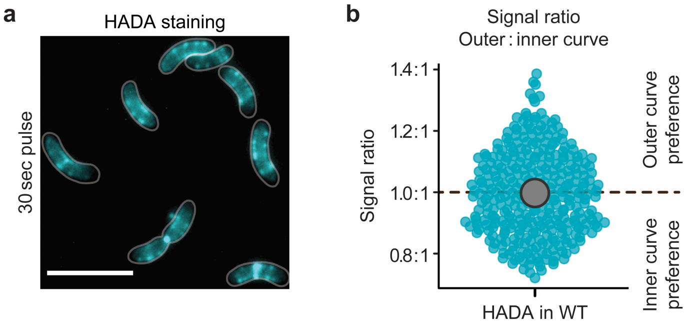

Supplementary Figure 12. HADA staining of *R. rubrum* cells. (a) Widefield epifluorescence image of representative *R. rubrum* wild-type (S1) cells after 30 sec of incubation with the D-amino acid analog HADA. The cell outlines are indicated in grey. **(b)** Outer-to-inner-curve HADA signal ratios in the cells analyzed in panel a (n=331 cells).

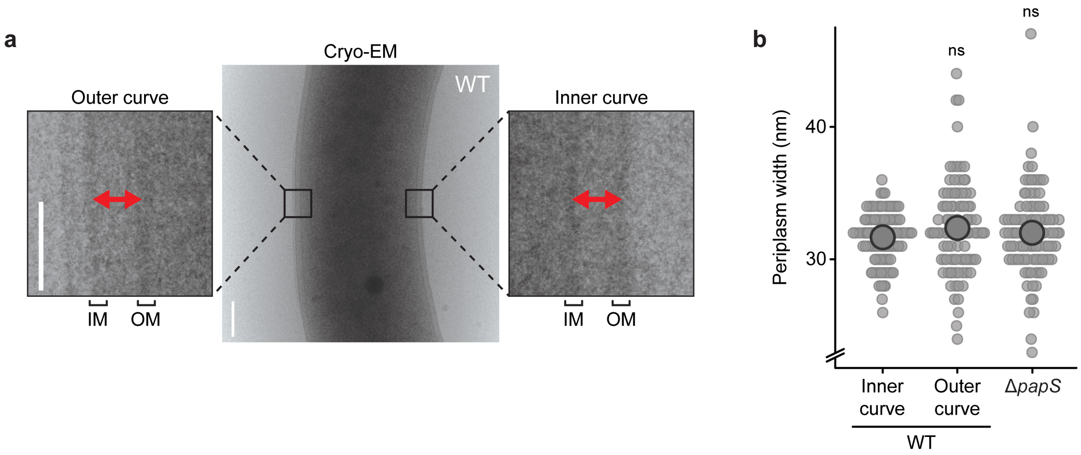

Supplementary Figure 13. Width of the periplasmic space in *R. rubrum* wild-type and Δ*papS* cells. (a) Cryo-electron tomogram of a representative *R. rubrum* wild-type cell (n=20 cells). The images on the left and right provide a magnified view of the periplasmic space at the outer and inner curve of the cell. The width of the periplasmic space is indicated by red left-right arrows. IM: inner membrane, OM: outer membrane. Bar: 50 nm. **(b)** Beeswarm plots showing a quantification of periplasm width at the inner (n=10 measurements per cells) and outer (n=10 measurements per cell) curve of *R. rubrum* wild-type (S1) cells and at a random lateral position in Δ*papS* cells (JR52). Small dots represent the data points, large dots represent the median values.

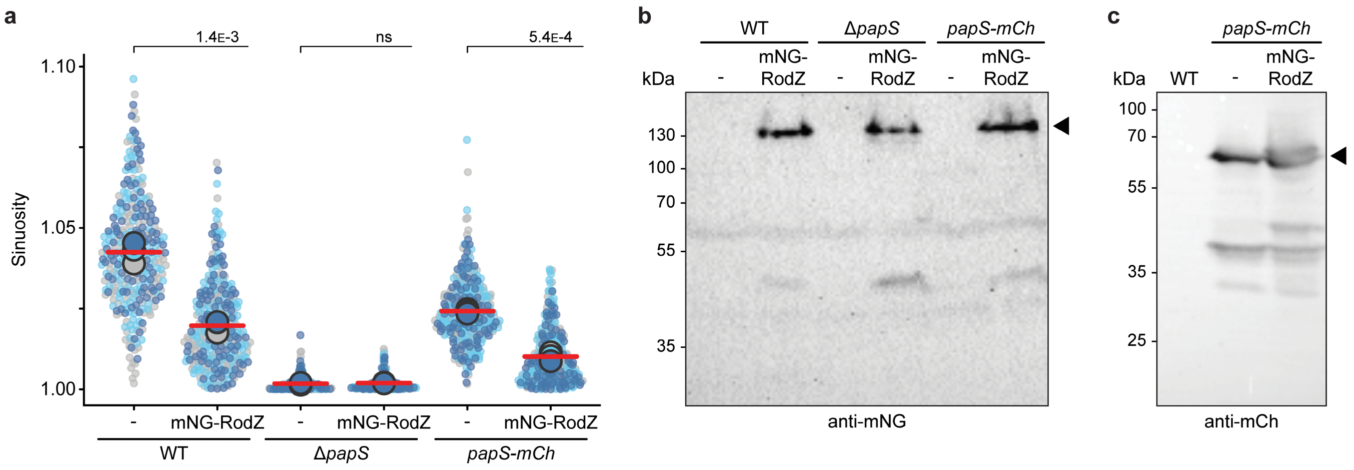

Supplementary Figure 14. Analysis of cells producing mNG-RodZ. (a) Superplots showing the distribution of cell sinuosities in populations of *R. rubrum* cells producing mNG-RodZ in place of the native RodZ protein in the wild-type (SP160), Δ*papS* (SP162) or *papS-mCh* (SP163) background. The sinuosity distributions of the wild-type (S1) (replotted from **Figure 1f**), Δ*papS* (JR52) and *papS-mCh* (SP01) parental strains are shown as a reference. The data are presented as described in **Supplementary Figure 1a** (n=100 cells per replicate). The statistical significance (*p* value) of differences between strains is indicated (unpaired two-sided Welch's t-test; ns, not significant). **(b)** Immunoblot analysis of the strains described in panel a. Proteins were detected with an anti-mNeonGreen antibody. The predicted molecular weight of mNG-RodZ is 79.1 kDa (indicated by an arrowhead). Note that mNG-RodZ shows an aberrant migration behavior in SDS gels. **(c)** Immunoblot analysis of *R. rubrum* wild-type (S1), *papS-mCh* (SP01) and *papS-mCh mNG-rodZ* (SP160) cells. Proteins were detected with an anti-mCherry antibody. The predicted molecular weight of processed PapS-mCh is 55.4 kDa (indicated by an arrowhead).

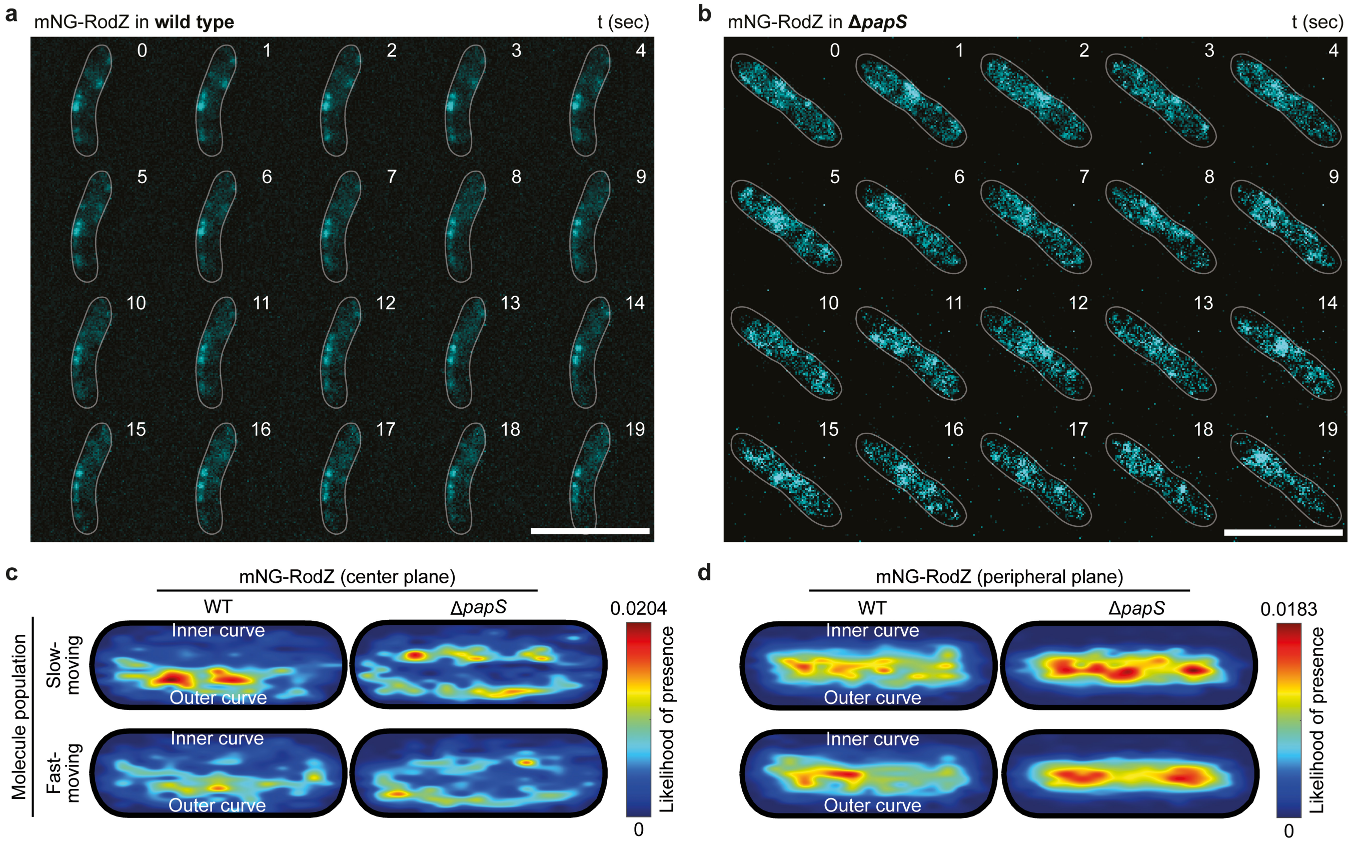

Supplementary Figure 15. Analysis of the dynamics of mNG-RodZ motion. (a,b) Montages showing the localization pattern of mNG-RodZ in the (a) wild-type (SP160) and (b) Δ*papS* (SP162) background over time, recorded at one-second intervals. Bar: 3 µm. Shown are the representative cells (n=3 biological replicates) used to generate the kymographs in **Figure 9**. **(c,d)** Confinement maps showing the likelihood of presence for slow-moving and fast-moving mNG-RodZ molecules in the wild-type (SP160) and Δ*papS* (SP162) backgrounds, as determined by the tracking of single mNG-RodZ particles at the (c) center or (d) peripheral plane of the cell. The slow-moving fraction was defined as molecules that stay within a radius of 97 nm for at least 0.12 ms. Wild-type cells were oriented such that their outer curve faced to the lower edge of the confinement maps.

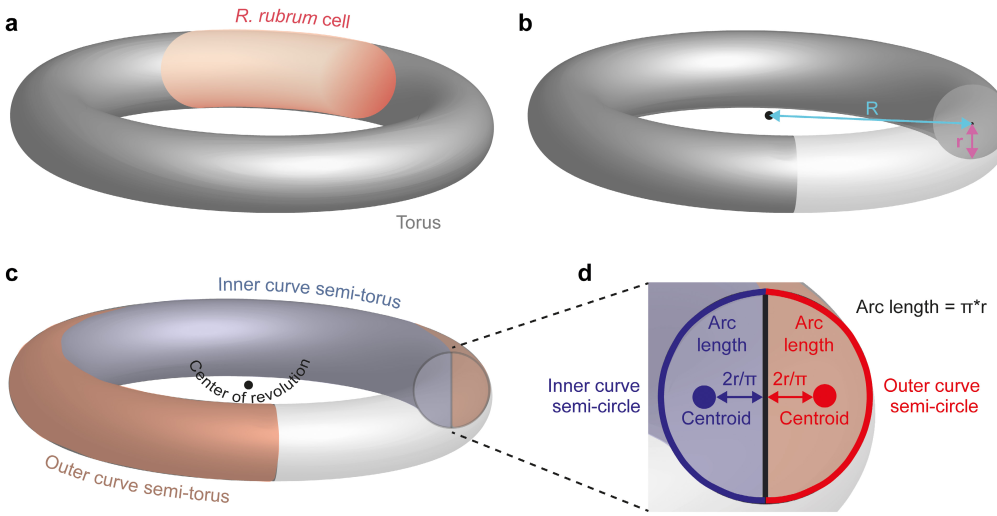

Supplementary Figure 16. Approximation of the difference in cell wall elongation rates at the inner and outer curves required to generate the typical curvature of *R. rubrum* cells. **(a)** An *R. rubrum* cell can be described by a fragment of a torus shape. **(b)** A torus is described by the outer radius R and the inner radius r. **(c)** A torus shape can be divided into an inner-curve semi-torus and an outer-curve semi-torus. The exposed surface areas of these shapes differ in size. **(d)** The surface areas of the inner and outer curves are described by Pappus' first centroid theorem, which relates the surface area of a solid of revolution (semi-circle) to the arc length of the generating curve and the distance traveled by the centroid of the curve.

Supplementary tables

Supplementary Table 1. *R. rubrum* strains used in this study.

Strain	Genotype/description	Construction	Reference/Source
S1	Wild type (aka ATCC 11170, DSM 467)	-	[4]
JR52	Δ*papS* (*Rru_A3328*)	In-frame deletion of *papS* in S1 using pJR85	This study
JR54	Δ*papS* P_{papS}-*papS*	Transformation of JR52 with pJR88	This study
JR56	Δ*papS* P_{papS}-*papS-mCherry*	Transformation of JR52 with pJR92	This study
LMS23	*papS::papS-mNeongreen por39::mCherry-por39*$_{(T280S\ P282S)}$	Chromosomal mutation of *por39* in SP06 using pSP181	This study
LMS24	*papS::papS-mNeongreen por39::mCherry-por39 por41::por41*$_{(V290S\ P292S)}$ (*Rru_A2211*)	Chromosomal mutation of *por41* in SP06 using pSP182	This study
SP01	*papS*::*papS-mCherry*	Replacement of *papS* with *papS-mCherry* in S1 using pSP01	This study
SP02	*papS*::*papS-mNeongreen*	Replacement of *papS* with *papS-mNeongreen* in S1 using pSP02	This study
SP04	*papS*::*papS-PAmCherry*	Replacement of *papS* with *papS-PAmCherry* in S1 using pSP04	This study
SP06	*papS*::*papS-mNeongreen por39* (*Rru_A2212*)::*mCherry-por39*	Replacement of *por39* with mCherry-*por39* in SP02 using pSP08	This study
SP12	Δ*papS por39*::*mCherry-por39*	Replacement of *por39* with mCherry-*por39* in JR52 using pSP08	This study
SP23	Δ*papS* pRXMCS-2	Transformation of JR52 with pRXMCS-2	This study
SP126	Δ*papS por39*::*mCherry-por39* P_{papS}-*papS-mNeongreen*	Transformation of SP12 with pSP138	This study
SP130	*papS*::*papS-mNeongreen mCherry-por39*::*mCherry-por39*$_{(D71S)}$	Chromosomal mutation of *por39* in SP06 using pSP135	This study
SP131	*papS*::*papS-mNeongreen por39*::*mCherry-por39 por41*::*por41*$_{(D71S)}$	Chromosomal mutation of *por41* in SP06 using pSP136	This study
SP136	Δ*papS* P_{papS}-*papS-mNeongreen*	Transformation of JR52 with pSP138	This study
SP137	Δ*papS* P_{papS}-*CCNA_02075*$_{1\text{-}28}$-*papS*$_{18\text{-}273}$-*mNeongreen*	Transformation of JR52 with pSP143	This study
SP143	Δ*papS por39*::*mCherry-por39* P_{papS}-*papS*$_{(W22A)}$-*mNeongreen*	Transformation of SP12 with pSP139	This study
SP144	Δ*papS por39*::*mCherry-por39* P_{papS}-*papS*$_{(W58A)}$-*mNeongreen*	Transformation of SP12 with pSP140	This study
SP145	Δ*papS* P_{papS}-*papS*$_{(R223A)}$-*mNeongreen*	Transformation of JR52 with pSP142	This study
SP146	Δ*papS por39*::*mCherry-por39* P_{papS}-*papS*$_{(W22A\ W58A)}$-*mNeongreen*	Transformation of SP12 with pSP141	This study
SP150	*por41*::*por41*$_{(D71S)}$	Chromosomal mutation of *por41* in S1 using pSP136	This study
SP160	*rodZ* (*Rru_A0746*)::*mNeongreen-rodZ*	Replacement of *rodZ* with *mNeongreen-rodZ* in S1 using pSP154	This study
SP162	Δ*papS rodZ*::*mNeongreen-rodZ*	Replacement of *rodZ* with *mNeongreen-rodZ* in JR52 using pSP154	This study
SP163	*papS*::*papS-mCherry rodZ*::*mNeongreen-rodZ*	Replacement of *rodZ* with *mNeongreen-rodZ* in SP01 using pSP154	This study
SP165	Δ*papS* P_{papS}-*papS-PAmCherry*	Transformation of JR52 with pSP155	This study
SP166	Δ*papS* P_{papS}-*CCNA_02075*$_{1\text{-}28}$-*papS*$_{18\text{-}273}$-*PAmCherry*	Transformation of JR52 with pSP156	This study
SP167	Δ*papS* P_{papS}-*papS*$_{(R223A)}$-*PAmCherry*	Transformation of JR52 with pSP157	This study
SP168	Δ*papS* P_{papS}-*papS*$_{(W22A\ W58A)}$-*PAmCherry*	Transformation of JR52 with pSP158	This study
SP173	*por39*::PAmCherry-*por39*	Replacement of *por39* with *PAmCherry-por39* in S1 using pSP159	This study
SP174	Δ*papS* P_{papS}-*papS*$_{(W22A\ W58A\ R223A)}$-*mNeongreen*	Transformation of JR52 with pSP164	This study
SP175	Δ*papS* P_{papS}-*papS*$_{(W22A\ W58A\ R223A)}$-*PAmCherry*	Transformation of JR52 with pSP165	This study
SP177	Δ*papS por39*::PAmCherry-*por39*	Replacement of *por39* with *PAmCherry-por39* in JR52 using pSP159	This study
SP183	*papS*::*papS-PAmCherry por41*::*por41*$_{(D71S)}$	Chromosomal mutation of *por41* inSP04 using pSP136	This study
SP184	Δ*papS* P_{papS}-*CCNA_02075*$_{1\text{-}28}$-*papS*$_{18\text{-}273}$	Transformation of JR52 with pSP171	This study
SP185	Δ*papS* P_{papS}-*papS*$_{(R223A)}$	Transformation of JR52 with pSP172	This study
SP186	Δ*papS* P_{papS}-*papS*$_{(W22A\ W58A)}$	Transformation of JR52 with pSP173	This study
SP187	Δ*papS* P_{papS}-*papS*$_{(W22A\ W58A\ R223A)}$	Transformation of JR52 with pSP174	This study
SP197	Δ*papS* P_{papS}-*papS*$_{(W22A\ W58A)}$-*mNeongreen*	Transformation of JR52 with pSP141	This study
SP215	*por39*::*por39*$_{(D71S)}$	Chromosomal mutation of *por39* in S1 using pSP178	This study
SP222	*por39*::*mCherry-por39*	Replacement of *por39* with mCherry-*por39* in S1 using pSP08	This study

Supplementary Table 1. ***R. rubrum*** **strains used in this study (continued).**

Strain	Genotype/description	Construction	Reference/Source
SP231	*papS*::*papS-bla*	Replacement of *papS* with *papS-bla* in S1 using pSP194	This study
SP234	*por41::por41*$_{(V290S\ P292S)}$	Chromosomal mutation of *por41* in S1 using pSP182	This study
SP235	*por39::por39*$_{(T280S\ P282S)}$	Chromosomal mutation of *por39* in S1 using pSP181	This study
SP242	*por39::por39*$_{(T280S\ P282S)}$ *por41::por41*$_{(V290S\ P292S)}$	Chromosomal mutation of *por39* in SP234 using pSP181	This study
SP243	*papS::papS-mNeongreen por39::mCherry-por39*$_{(T280S\ P282S)}$ *por41::por41*$_{(V290S\ P292S)}$	Chromosomal mutation of *por39* in LMS24 using pSP181	This study

Supplementary Table 2. ***E. coli*** **strains used in this study.**

Strain	Genotype/description	Source
SHuffle T7	F´ *lac, pro, lacI*^{q} / Δ(*ara-leu*)7697 *araD*139 *fhuA*2 *lacZ*::T7 gene1 Δ(*phoA*)PvuII *phoR ahpC** *galE* (or *U*) *galK* λ*att*::pNEB3-r1-cDsbC (Spec^R, *lacI*^{q}) Δ*trxB rpsL*150(Str^R) Δ*gor* Δ(*malF*)3	New England Biolabs
TOP10	F⁻ *mcrA* Δ(*mrr-hsd*RMS-*mcr*BC) Φ80*lac*ZΔM15 Δ*lac*X74 *recA*1 *araD*139 Δ(*ara-leu*) 7697 *galU galK rpsL* (Str^R) *endA*1 *nup*G	Thermo Fisher Scientific
Rosetta(DE3)pLysS	F$^-$ *ompT hsdS*$_B$(r_B^- m_B^-) *gal dcm* (DE3) pLysSRARE (Cam^R)	Merck Millipore
WM3064	*thrB1004 pro thi rpsL hsdS lacZ*ΔM15 RP4–1360 Δ(*araBAD*)567 Δ*dapA*1341::[*erm pir*(wt)]	W. Metcalf (unpublished)

Supplementary Table 3. Plasmids used in this work.

Plasmid	Description	Construction/Reference/Source
pAM142	pET28a(+) derivative carrying *malE-his*$_6$	[5]
pCHYC-2	Plasmid for the generation of C-terminal mCherry fusions, Kan^R	[6]
pJR85	pNPTS138 derivative for in-frame deletion of *papS*	a) amplification of the *papS* (*Rru_A3328*) flanking regions from S1 chromosomal DNA using primers oJR58 + oJR59 and oJR60 + oJR61<br>b) restriction of fragment 1 with EcoRI/HindIII and fragment 2 with EcoRI/NheI<br>c) triple ligation with pNPTS138 cut with HindIII/NheI
pJR86	pCHYC-2 derivative carrying P$_{papS}$-*papS-mCherry*	a) amplification of P$_{papS}$-*papS* from S1 chromosomal DNA using primers oJR56 + oJR57<br>b) restriction of the fragment with NdeI/SacI<br>c) ligation with pCHYC-2 cut with NdeI/SacI
pJR87	pTB146 derivative for the overproduction of His$_6$-SUMO-PapS$_{(AA\ 18\text{-}273)}$	a) amplification of *papS*$_{(AA\ 18\text{-}273)}$ with primers oJR74 and oJR75<br>b) restriction of the fragment with SapI and BamHI<br>c) ligation with pTB146 cut with SapI and BamHI
pJR88	pRXMCS-2 derivative carrying P$_{papS}$-*papS*	a) amplification of P$_{papS}$-*papS* from S1 chromosomal DNA using primers oJR72 + oJR73<br>b) restriction of the fragment with NotI/EcoRI<br>c) ligation with pRXMCS-2 cut with NotI/EcoRI
pJR92	pRXMCS-2 derivative carrying P$_{papS}$-*papS-mCherry*	a) Isolation of a fragment containing P$_{papS}$-*papS-mCherry* from pJR86 with SapI/NheI<br>c) ligation with pJR88 cut with SapI/NheI
pJR94	pRXMCS-2 derivative carrying P$_{papS}$-*CCNA_02075*$_{(AA\ 1\text{-}28)}$-*papS*$_{(AA\ 18\text{-}273)}$-*mCherry*	a) amplification of *CCNA_02075*$_{(AA\ 1\text{-}28)}$ from a synthetic DNA fragment using primers oJR81 + oJR82<br>b) restriction of the fragment with NotI/BstXI<br>c) ligation with pJR92 cut with NotI/BstXI
pmNeonGreen-CT	Plasmid containing mNeonGreen	Allele Biotech
pNPTS138	*sacB*-containing suicide vector used for double homologous recombination, Kan^R	M. R. K. Alley, unpublished
pRXMCS-2	Replicative low-copy number plasmid for the ectopic expression of genes under the control of P$_{xyl}$, Kan^R	[6]
pSP01	pNPTS138 derivative for the replacement of *papS* with *papS-mCherry*	a) amplification of the regions flanking the *mCherry* integration site from S1 chromosomal DNA using primers oSP001 + oSP523 and oSP524 + oSP004<br>b) amplification of *linker-mCherry* from pCHYC-2 using primers oSP005 + oSP006<br>c) insertion of both fragments into pNPTS138 cut with HindIII/NheI by Gibson assembly
pSP02	pNPTS138 derivative for the replacement of *papS* with *papS-mNeongreen*	a) amplification of the regions flanking the *mNeongreen* integration site from pSP01 using primers oSP009 + oSP010 and oSP013 + oSP014<br>b) amplification of *mNeongreen* from pmNeonGreen-CT using primers oSP011 + oSP012<br>c) insertion of both fragments into pNPTS138 cut with HindIII/NheI by Gibson assembly
pSP04	pNPTS138 derivative for native replacement of *papS* with *papS-PAmCherry*	a) amplification of the regions flanking the *PAmCherry* integration site from pSP01 using primers oSP009 + oSP002 and oSP022 + oSP014<br>b) amplification of *linker-PAmCherry* from a synthetic DNA fragment using primers oSP005 + oSP021<br>c) insertion of both fragments into pNPTS138 cut with HindIII/NheI by Gibson assembly
pSP08	pNPTS138 derivative for the replacement of *por39* with *mCherry-por39*	a) amplification of the regions flanking the *mCherry* integration site from S1 chromosomal DNA using primers oSP024 + oSP040 and oSP043 + oSP044<br>b) amplification of *mCherry-linker* from pXCHYN-2 using primers oSP041 + oSP042<br>c) insertion of both fragments into pNPTS138 cut with HindIII/NheI by Gibson assembly
pSP74	pTB146 derivative carrying *papS*$_{(AA156\text{-}273)}$	a) amplification of *papS*$_{(AA156\text{-}273)}$ from S1 chromosomal DNA using primers oSP303 + oSP222<br>b) insertion of the fragment into pTB146 cut with SapI/BamHI by Gibson assembly
pSP78	pTB146 derivative carrying *papS*$_{R223A(AA156\text{-}273)}$	a) Site-directed mutagenesis of pSP74 using primers oSP065 + oSP066
pSP134	pNPTS138 derivative for the replacement of *rodZ* with *yfp-rodZ*	a) amplification of the regions flanking the *yfp* integration site from S1 chromosomal DNA using primers oSP502 + oSP503 and oSP505 + oSP506<br>b) amplification of *yfp-linker* from pXYFPN-2 using primers oSP497 + oSP490<br>c) insertion of both fragments into pNPTS138 cut with HindIII/NheI by Gibson assembly
pSP135	pNPTS138 derivative for the generation of the *por39*$_{D71S}$ allele in SP06	a) amplification of the regions flanking the D71 codon of *por39* from SP06 chromosomal DNA using primers oSP509 + oSP510 and oSP511 + oSP512. The amplification products contain the desired mutation.<br>b) insertion of both fragments into pNPTS138 cut with HindIII/NheI by Gibson assembly

Supplementary Table 3. Plasmids used in this work (continued).

Plasmid	Description	Construction/Reference/Source
pSP136	pNPTS138 derivative for the generation of the *por41*$_{D71S}$ allele	a) amplification of the regions flanking the D71 codon of *por41* from SP06 chromosomal DNA using primers oSP515 + oSP516 and oSP517 + oSP518. The amplification products contain the desired mutation.<br>b) insertion of both fragments into pNPTS138 cut with HindIII/NheI by Gibson assembly
pSP138	pRXMCS-2 derivative carrying P$_{papS}$-*papS-mNeongreen*	a) amplification of P$_{papS}$-*papS* from S1 chromosomal DNA using primers oLH001 + oSP523<br>b) amplification of *mNeongreen* from pSP02 using primers oSP524 + oSP525<br>c) insertion of both fragments into pRXMCS-2 cut with NotI/EcoRI by Gibson assembly
pSP139	pSP138 derivative carrying P$_{papS}$-*papS*$_{W22A}$-*mNeongreen*	a) Site-directed mutagenesis of pSP138 using primers oSP521 + oSP522
pSP140	pSP138 derivative carrying P$_{papS}$-*papS*$_{W58A}$-*mNeongreen*	a) Site-directed mutagenesis of pSP138 using primers oSP526 + oSP527
pSP141	pSP139 derivative carrying P$_{papS}$-*papS*$_{W22A\ W58A}$-*mNeongreen*	a) Site-directed mutagenesis of pSP138 using primers oSP526 + oSP527
pSP142	pSP138 derivative carrying P$_{papS}$-*papS*$_{R223A}$-*mNeongreen*	a) Site-directed mutagenesis of pSP138 using primers oSP065 + oSP066
pSP143	pRXMCS-2 derivative carrying P$_{papS}$-*CCNA_02075*$_{1-28}$-*papS*$_{18-273}$-*mNeongreen*	a) amplification of P$_{papS}$-*CCNA_02075*$_{1-28}$-*papS*$_{18-273}$ from pJR94 using primers oLH001 + oSP523<br>b) amplification of *mNeongreen* from pSP02 using primers oSP524 + oSP525<br>c) insertion of both fragments into pRXMCS-2 cut with NotI/EcoRI by Gibson assembly
pSP154	pNPTS138 derivative for the replacement of *rodZ* with *mNeongreen-rodZ*	a) amplification of the regions flanking the *mNeongreen* integration site from pSP134 using primers oSP502 + oSP563 and oSP566 + oSP506<br>b) amplification of *mNeongreen* from pSP02 using primers oSP564 + oSP565<br>c) insertion of the three fragment into pNPTS138 cut with HindIII/NheI by Gibson assembly
pSP155	pRXMCS-2 derivative carrying P$_{papS}$-*papS-PAmCherry*	a) amplification of P$_{papS}$-*papS* from S1 chromosomal DNA using primers oLH001 + oSP523<br>b) amplification of *linker-PAmCherry* from pSP04 using primers oSP524 + oLH025<br>b) insertion of both fragments into pRXMCS-2 cut with NotI/EcoRI by Gibson assembly
pSP156	pRXMCS-2 derivative carrying P$_{papS}$-*CCNA_02075*$_{1-28}$-*papS*$_{18-273}$-*PAmCherry*	a) amplification of P$_{papS}$-*CCNA_02075*$_{1-28}$-*papS*$_{18-273}$ from pJR94 using primers oLH001 + oSP523<br>b) amplification of *linker-PAmCherry* from pSP04 using primers oSP524 + oLH025<br>b) insertion of both fragments into pRXMCS-2 cut with NotI/EcoRI by Gibson assembly
pSP157	pRXMCS-2 derivative carrying P$_{papS}$-*papS*$_{R223A}$-*PAmCherry*	a) amplification of P$_{papS}$-*papS*$_{R223A}$ from pSP142 using primers oLH001 + oSP523<br>b) amplification of *linker-PAmCherry* from pSP04 using primers oSP524 + oLH025<br>b) insertion of both fragments into pRXMCS-2 cut with NotI/EcoRI by Gibson assembly
pSP158	pRXMCS-2 derivative carrying P$_{papS}$-*papS*$_{W22A\ W58A}$-*PAmCherry*	a) amplification of P$_{papS}$-*papS*$_{W22A\ W58A}$ from SP141 using primers oLH001 + oSP523<br>b) amplification of *linker-PAmCherry* from pSP04 using primers oSP524 + oLH025<br>b) insertion of both fragments into pRXMCS-2 cut with NotI/EcoRI by Gibson assembly
pSP159	pNPTS138 derivative for the replacement of *por39* with *PAmCherry-por39*	a) amplification of the regions flanking the PA*mCherry* integration site from pSP08 using primers oSP024 + oSP040 and oSP568 + oSP044<br>b) amplification of PA*mCherry* from pSP04 using primers oSP041 + oSP567<br>c) insertion of both fragments into pNPTS138 cut with HindIII/NheI by Gibson assembly
pSP164	pSP141 derivative carrying P$_{papS}$-*papS*$_{W22A\ W58A\ R223A}$-*mNeongreen*	a) Site-directed mutagenesis of pSP141 using primers oSP065 + oSP066
pSP165	pSP158 derivative carrying P$_{papS}$-*papS*$_{W22A\ W58A\ R223A}$-*PAmCherry*	a) Site-directed mutagenesis of pSP158 using primers oSP065 + oSP066
pSP171	pRXMCS-2 derivate carrying P$_{papS}$-*CCNA_02075*$_{(AA\ 1-28)}$-*papS*$_{18-273}$	a) amplification of P$_{papS}$-*CCNA_02075*$_{1-28}$-*papS*$_{18-273}$ from pSP156 using primers oLH001 + oSP347<br>b) insertion of the fragment into pRXMCS-2 cut with NotI/EcoRI by Gibson assembly
pSP172	pRXMCS-2 derivative carrying P$_{papS}$-*papS*$_{R223A}$	a) amplification of P$_{papS}$-*papS*$_{R223A}$ from pSP157 using primers oLH001 + oSP347<br>b) insertion of the fragment into pRXMCS-2 cut with NotI/EcoRI by Gibson assembly
pSP173	pRXMCS-2 derivative carrying P$_{papS}$-*papS*$_{W22A\ W58A}$	a) amplification of P$_{papS}$-*papS*$_{W22A\ W58A}$ from pSP158 using primers oLH001 + oSP347<br>b) insertion of the fragment into pRXMCS-2 cut with NotI/EcoRI by Gibson assembly
pSP174	pSP158 derivative carrying P$_{papS}$-*papS*$_{W22A\ W58A\ R223A}$	a) amplification of P$_{papS}$-*papS*$_{W22A\ W58A\ R223A}$ from pSP165 using primers oLH001 + oSP347<br>b) insertion of the fragment into pRXMCS-2 cut with NotI/EcoRI by Gibson assembly
pSP178	pNPTS138 derivative for the generation of the *por39*$_{D71S}$ allele in S1	a) amplification of the regions flanking the D71 codon from S1 chromosomal DNA using primers oSP616 + oSP510 and oSP511 + oSP512. The amplification products contain the desired mutation.<br>b) insertion of both fragments into pNPTS138 cut with HindIII/NheI by Gibson assembly
pSP186	pRXMCS-2 derivative carrying P$_{papS}$-*papS-bla*	a) amplification of P$_{papS}$-*papS* from S1 chromosomal DNA using primers oLH001 + oSP649<br>b) amplification of *linker-bla* from pXBlaMC-2 using primers oSP650 + oSP651<br>b) insertion of both fragments into pRXMCS-2 cut with NotI/EcoRI by Gibson assembly

Supplementary Table 3. Plasmids used in this work (continued).

Plasmid	Description	Construction/Reference/Source
pSP194	pNPTS138 derivative for the replacement of *papS* with *papS-bla*	a) amplification of the upstream flanking region of the *bla* integration site and of *bla* from pSP186 using primers oSP676 + oSP677<br>b) amplification of the downstream flanking region of the *bla* integration site from S1 chromosomal DNA using primers oSP678 + oSP679<br>b) insertion of both fragments into pNPTS138 cut with HindIII/NheI by Gibson assembly
pSP213	pTB146 derivative carrying *Acinetobacter baumannii ompA*$_{(AA221\text{-}339)}$	Insertion of a synrthetic *Acinetobacter baumannii ompA*$_{(AA221\text{-}339)}$ gene fragment (Genscript, USA) into pTB146 cut with SapI/BamHI by Gibson assembly
pTB146	Plasmid for the overproduction of N-terminally His_6-SUMO-tagged proteins, Amp^R	[7]
pXBlaMC-2	Plasmid for the construction of C-terminal β-lactamase fusions, Kan^R	[5]
pXYFPN-2	Integrating plasmid for the generation of N-terminal YFP fusions encoded at the *xylX*-locus, Kan^R	[6]

Supplementary Table 4. **PCR primers used in this work.**

ID	Sequence
oJR56	TTTTCATATGTACACCAACAAGGCGAAGCTGGCCG
oJR57	TTGAGCTCGGCCGCCGAAGACCACTTCGACACGAC
oJR58	TTAAGCTTCGATCGCCTTGGCGACGTTGTCGTAG
oJR59	TTGAATTCGGCAACGCTGATCAGCACCTTATGCATCAT
oJR60	TTGAATTCGAGCAGCAGAACCGTCGTGTCGAAGTG
oJR61	TTGCTAGCCGGTCACCAAGGTGACGGCGATGAAG
oJR72	TTGCGGCCGCAGCGCTTTCTTTGCTCCGGGACCT
oJR73	TTGAATTCTTAGCCGCCGAAGACCACTTCGACAC
oJR74	CTCACAGAGAACAGATTGGTGGTGCCGACGTATGGAACTACGAGG
oJR75	TCGGGCTTTGTTAGCAGCCGTTAGCCGCCGAAGACCACTT
oJR81	CGAGGAAGCGGCCGCAGCGCTTTCTTTG
oJR82	CTCGGCCATCGCCGTGGGGGCAGGGGTC
oLH001	CAGCGAGTCAGTGAGCGAGGAAGCCAGCGCTTTCTTTGCTCCGGGAC
oLH025	GCTCACCACGTGGTACCTCGAGTTACTTGTACAGCTCGTCCATGCCGCC
oSP001	CAATTGAAGCCGGCTGGCGCCAAGCTTGTGGCTACGCAGAGCAATGCGG
oSP002	CCGGTGAAGGAAAACGCGGAGCCTTACTTGTACAGCTCGTCCATGCCGC
oSP004	GAGACGCGTCACGGCCGAAGCTAGCCCGGTCACCAAGGTGACGGC
oSP005	CGGGATTGCCGCAGTTTCCCAG
oSP006	GGGTGCCTGATCTTCGTGGCG
oSP009	CAATTGAAGCCGGCTGGCGCCAAGCTTGGCCAGACCCCTGCCCCCAC
oSP010	CATGTTATCCTCCTCGCCCTTGCTCACCATGGTGGCCGACCGGTGACGC
oSP011	GTTACGCGTCACCGGTCGGCCACCATGGTGAGCAAGGGCGAGGAGG
oSP012	CACCGGTGAAGGAAAACGCGGAGCCTTACTTGTACAGCTCGTCCATGCC
oSP013	GGCATGGACGAGCTGTACAAGTAAGGCTCCGCGTTTTCCTTCACCG
oSP014	CCGGAGACGCGTCACGGCCGAAGCTAGCGGAACGGCGATGGGCATCG
oSP021	CCGGTGAAGGAAAACGCGGAGCCTTACTTGTACAGCTCGTCCATGCCGC
oSP022	GGCGGCATGGACGAGCTGTACAAGTAAGGCTCCGCGTTTTCCTTCACCG
oSP024	CAATTGAAGCCGGCTGGCGCCAAGCTTGTTCGCCCTGGCCTTTCCCAAG
oSP040	CCTCCTCGCCCTTGCTCACCATCGAGATCGGATCGGCGGCCAC
oSP041	CCGTGGCCGCCGATCCGATCTCGATGGTGAGCAAGGGCGAGGAGG
oSP042	GGACAGCGAGATCGGATCGGCCGGAGCTCGAGATCTTAAGGTACCATGC
oSP043	GGTACCTTAAGATCTCGAGCTCCGGCCGATCCGATCTCGCTGTCCTTG
oSP044	GGAGACGCGTCACGGCCGAAGCTAGCCAGGCCGCCCTGGTAGATCCAG
oSP065	CATCTTGTTGTCGCAGCGTGCCGCCGAAGCC
oSP066	GGCTTCGGCGGCACGCTGCGACAACAAGATG
oSP222	CCTTTCGGGCTTTGTTAGCAGCCGTTAGCCGCCGAAGACCACTTCGAC
oSP303	GAGGCTCACAGAGAACAGATTGGTGGTGGCGCTCCGGCCCCG
oSP347	GCTCACCACGTGGTACCTCGAGTTAGCCGCCGAAGACCACTTCGACAC
oSP490	CGTTCCGCCTCCGGATCCGCCTCCCTTGTACAGCTCGTCCATGCCGAG
oSP497	CGGGGTAGCGGAGTAAAGACGCATGGTGAGCAAGGGCGAGGAGC
oSP502	GCAATTGAAGCCGGCTGGCGCCAGGAATTGCGCGTCATGTTCCCGATG
oSP503	CAGCTCCTCGCCCTTGCTCACCATGATGTCCTCATTCCAGCGCGGATCC
oSP505	GAGGCGGATCCGGAGGCGGAACGTTGAAGACATCCGTTCCCGACACCTC
oSP506	CATCCGGAGACGCGTCACGGCCGAAGTGCGAGACCAGCACCTCGGGC
oSP509	GCAATTGAAGCCGGCTGGCGCCAGGACATCCTGTCCCCTCAGTTCATG
oSP510	GCCGTTCGACAAGGTCGTGCTGCCCTTGAAATAGACCTCGGTATCGGTG
oSP511	CTATTTCAAGGGCAGCACGACCTTGTCGAACGGCATCACCGTTGCCG
oSP512	CCGGAGACGCGTCACGGCCGAAGGCTTGTAGATGGCATTGTCGCCACCG
oSP515	GCAATTGAAGCCGGCTGGCGCCACAGCGGCACCGTCTATTCGACC
oSP516	GCCGTTCGACAAGGTCGTGCTACCGGAGAAGTACACTTCGGTGTCG
oSP517	CTTCTCCGGTAGCACGACCTTGTCGAACGGCATCACCGTCGCCG
oSP518	CATCCGGAGACGCGTCACGGCCGAAGCATCCACACCGGACTTGCTGTCG
oSP521	GCCTGCGCCGACGTAGCGAACTACGAGGAAGTGG
oSP522	CCACTTCCTCGTAGTTCGCTACGTCGGCGCAGGC
oSP523	CGTTCGAATTCTCCGGAGCTCGGCCGCCGAAGACCACTTCGAC
oSP524	CGAAGTGGTCTTCGGCGGCCGAGCTCCGGAGAATTCGAACGTTACG
oSP525	CACCACGTGGTACCTCGAGTTACTTGTACAGCTCGTCCATGCCCATCAC
oSP526	CGAAGCGAACTATGGCGATGCGGACGATACCGCCTATTACAC
oSP527	GTGTAATAGGCGGTATCGTCCGCATCGCCATAGTTCGCTTCG
oSP563	CCTCCTCGCCCTTGCTCACCATGATGTCCTCATTCCAGCGCGGATCC
oSP564	GGATCCGCGCTGGAATGAGGACATCATGGTGAGCAAGGGCGAGGAGG
oSP565	CCGCCTCCGGATCCGCCTCCCTTGTACAGCTCGTCCATGCCCATCAC

Supplementary Table 4. PCR primers used in this work (continued).

ID	Sequence
oSP566	GATGTGATGGGCATGGACGAGCTGTACAAGGGAGGCGGATCCGGAGGCG
oSP567	GCATATTAATTAAGGCGCCTGCAGGCTTGTACAGCTCGTCCATGCCGCC
oSP568	GGACGAGCTGTACAAGCCTGCAGGCGCCTTAATTAATATGCATGGTAC
oSP616	GCAATTGAAGCCGGCTGGCGCCAACGAATACCGCCACGATCTGGTTTCG
oSP627	CGTGCAATTGAAGCCGGCTGGCGCCAGTCCTTGGGCGGTAAGCAGGAG
oSP628	CAACCGAATACGACCCCGACGCGTAGCTGAGGCCGGCG
oSP629	CGCGTCGGGGTCGTATTCGGTTGGCTTCGCCTACGCCCAG
oSP630	CCGGAGACGCGTCACGGCCGAAGACGCTGACCGGCTTCGATCTTACC
oSP633	GTGCAATTGAAGCCGGCTGGCGCCACGCCTCCACGAAGGACGGTG
oSP634	GACCGAATACGACCCCGACGCATAGGACAGACCAGCGGTCCAC
oSP635	GCGTCGGGGTCGTATTCGGTCGGCTTCACCTACGGCCAGAC
oSP636	CATCCGGAGACGCGTCACGGCCGAAGCCATCGGCCGTCCCTCCAGATC
oSP649	CGGTGACGCGTAACGTTCGAATTCGCGCCGCCGAAGACCACTTCGACAC
oSP650	CGAAGTGGTCTTCGGCGGCGCGAATTCGAACGTTACGCGTCACCGGTC
oSP651	GCTCACCACGTGGTACCTCGAGTTACCAATGCTTAATCAGTGAGGCACC
oSP676	GCAATTGAAGCCGGCTGGCGCCATCAGCGTTGCCGCCGCC
oSP677	CGCGGAGCCTTACCAATGCTTAATCAGTGAGGCACCTATCTCAGC
oSP678	GGTGCCTCACTGATTAAGCATTGGTAAGGCTCCGCGTTTTCCTTCACCG
oSP679	CATCCGGAGACGCGTCACGGCCGAAGTCGATTTCTCCAGCCTGACGCCC

Supplementary Note 1

Based on our hypothesis that the curved cell shape of *R. rubrum* is the result of a stimulation of peptidoglycan biosynthesis in the outer curve, we aimed to mathematically quantify the relative increase in cell elongation in the outer curve required to generate the cell curvature observed. To this end, we sought to determine the percentage difference in the surface areas of the inner-curve and outer-curve half-cells.

To this end, we used a torus shape as a mathematical approximation model, assuming that an *R. rubrum* cell can be represented by a fraction of this shape (**Supplementary Figure 16a**). The torus has an outer radius R and an inner radius r (**Supplementary Figure 16b**). It can be divided into two semi-tori, namely the inner-curve and outer-curve semi-torus, by cutting it along the wall of a cylinder with a radius R (**Supplementary Figure 16c**). The surface area of the inner and outer curves can be calculated using Pappus' first centroid theorem, which relates the surface area of a solid of revolution to the arc length of the generating curve and the distance traveled by the centroid of the curve. The theorem states that the surface area A of the solid of revolution is equal to the product of the arc length of the generating curve s and the distance d traveled by the centroid of the curve during the revolution.

$$A(solid\ of\ revolution) = s(generating\ curve) * d(distance\ of\ centroid\ travel)$$

A solid of revolution is a three-dimensional object that is created by rotating a two-dimensional shape, called the generating curve, around a center of revolution. In our approximation model, the solids of revolution are the two semi-tori that are created by rotating two semi-circles, which are the generating curves (**Supplementary Figure 16c**). The center of revolution is positioned at the center of mass of the torus.

The arc length s of a semi-circle is defined as half the circumference of the corresponding full-circle (**Supplementary Figure 16d**).

$$s(semi\ circle) = \frac{2\pi r}{2} = \pi r$$

The centroid of a generating curve is the geometric center of its area. For a semi-circle arc, the centroid is defined to be at $\frac{2r}{\pi}$ distance from the flat side of the semi-circle. The distance of the inner-curve semi-circle arc centroid from the center of revolution can be calculated by subtracting the centroid position from the radius R that describes the distance of the full-torus center from the center of revolution (**Supplementary Figure 16b**).

$$Centroid(inner\ curve\ semi\ circle\ arc) = (R - \frac{2r}{\pi})$$

For the outer-curve semi-circle arc, the centroid position is added to the radius of the full-torus center.

$$Centroid(outer\ curve\ semi\ circle\ arc) = (R + \frac{2r}{\pi})$$

The distance traveled by the centroid during the revolution is described as the circumference of the circular path that the centroid follows during revolution.

$$d(centroid\ inner\ curve\ semi\ circle\ arc) = 2\pi(R - \frac{2r}{\pi})$$

$$d(centroid\ outer\ curve\ semi\ circle\ arc) = 2\pi(R + \frac{2r}{\pi})$$

The combination of these equations according to Pappus' theorem results in the following equations to calculate the inner and outer semi-torus curve surface areas:

$$A(inner\ semi\ torus) = 2\pi(R - \frac{2r}{\pi})\pi r$$

$$A(outer\ semi\ torus) = 2\pi(R + \frac{2r}{\pi})\pi r$$

To calculate the percentage difference between the surface areas of the inner and outer semi-tori, we can use the equation:

$$\% \, difference = \left| \frac{A(outer\ semi\ torus) - A(inner\ semi\ torus)}{A(inner\ semi\ torus)} \right| * 100$$

$$= \left| \frac{2\pi\left(R + \frac{2r}{\pi}\right)\pi r - 2\pi\left(R - \frac{2r}{\pi}\right)\pi r}{2\pi\left(R - \frac{2r}{\pi}\right)\pi r} \right| * 100$$

$$= \frac{4|r|}{|\pi R - 2r|} * 100$$

$$= \frac{400|r|}{|\pi R - 2r|}$$

Since an *R. rubrum* cell is represented by only a fraction of the torus, we need to identify the extent of this fraction. Based on microscopy data, we know that an average *R. rubrum* cell measures 3.46 ± 0.68 µm in length and 0.74 ± 0.05 µm in diameter. The poles of a cell are ~0.5 µm apart. The cell diameter dia gives the inner radius $r = \frac{dia}{2} = 0.37\ \mu m$. The outer radius R can be obtained from the mean curvature K of a cell, which is 0.29 ± 0.06 µm^{-1}. Given that $R = \frac{1}{K}$, the outer radius R is 3.47 µm.

In order to estimate the total surface area of an average *R. rubrum* cell, we aimed to determine the fraction of the torus that best describes an average cell. To do so, we calculated the circumference C of a torus that has the same outer radius R as a *R. rubrum* cell.

$$C = 2\pi R = 2\pi * 3.47\ \mu m = 21.79\ \mu m$$

Knowing that an average *R. rubrum* cell has a length of 3.46 µm and excluding the cell pole regions from the approximation, since they follow different geometrical rules and are not crucial for our approximation purposes, we can assume that an average cell corresponds to a torus fraction of

$$\frac{Cell\ length - 2 * Pole\ length}{C} * 100 = \frac{3.46\ \mu m - 2 * 0.5\ \mu m}{21.79\ \mu m} * 100 = 11.24\%$$

Using the equation for the calculation of the surface area of a torus $A(Torus) = 2\pi R 2\pi r$ and knowing that an *R. rubrum* cell is best described by a fraction of 11.24% of this torus, we can calculate the surface area of an average *R. rubrum* cell (excluding the poles):

$$A(Cell) = 2\pi R 2\pi r * 0.1124 = 2\pi * 3.47 \mu m * 2\pi * 0.37\ \mu m * 0.1124 = 5.71\ \mu m^2$$

The inner- and outer-curve semi-cell surface areas of an *R. rubrum* cell are

$$A(inner\ curve) = 2\pi\left(R - \frac{2r}{\pi}\right)\pi r * 0.1124 = 2\pi\left(3.47 \mu m - \frac{2 * 0.37\ \mu m}{\pi}\right)\pi * 0.37\ \mu m * 0.1124 = 2.66\ \mu m^2$$

$$A(outer\ curve) = 2\pi\left(R + \frac{2r}{\pi}\right)\pi r * 0.1124 = 2\pi\left(3.47 \mu m + \frac{2 * 0.37\ \mu m}{\pi}\right)\pi * 0.37\ \mu m * 0.1124 = 3.05\ \mu m^2$$

The percentage difference between inner and outer-curve semi-cell surface areas amounts to

$$\% \, difference = \frac{400|r|}{|\pi R - 2r|} = \frac{400 * |0.37\ \mu m|}{|\pi * 3.47 \mu m - 2 * 0.37 \mu m|} = 14.60\%$$

According to our model, the total amount of peptidoglycan incorporated in the outer curve needs to be 14.6% higher than that incorporated at the inner curve to generate the typical curvature of an average *R. rubrum* cell. This rather small difference in the extent of cell wall biosynthesis could explain why it was not possible to detect significant differences in the degree of HADA labeling at the inner and outer curves (compare **Supplementary Figure 12a,b**). Notably, the enrichment of mNG-RodZ in the outer curve observed in widefield fluorescence microscopy (compare **Figure 8c**) appears to be much higher than this value, suggesting that the activity of elongasomes is markedly reduced by their entrapment within the Porin-PapS structures, potentially due to a reduction in their processivity and significant idle times between changes in their direction of movement.

Supplementary References

1. Park J. S., Lee W. C., Yeo K. J., Ryu K. S., Kumarasiri M., Hesek D., Lee M., Mobashery S., Song J. H., Kim S. I., Lee J. C., Cheong C., Jeon Y. H., Kim H. Y. Mechanism of anchoring of OmpA protein to the cell wall peptidoglycan of the gram-negative bacterial outer membrane. *FASEB Journal* **26**, 219-228 (2012).
2. Jumper J*., et al.* Highly accurate protein structure prediction with AlphaFold. *Nature* **596**, 583-589 (2021).
3. Evans R*., et al.* Protein complex prediction with AlphaFold-Multimer. *bioRxiv* (2022). doi: 10.1101/2021.10.04.463034
4. Molisch H. *Die Purpurbakterien nach neuen Untersuchungen*. Gustav Fischer, Jena, pp. 1-95 (1907).
5. Möll A., Schlimpert S., Briegel A., Jensen G. J. & Thanbichler M. DipM, a new factor required for peptidoglycan remodelling during cell division in *Caulobacter crescentus*. *Molecular Microbiology* **77**, 90-107 (2010).
6. Thanbichler M., Iniesta A. A. & Shapiro L. A comprehensive set of plasmids for vanillate- and xylose-inducible gene expression in *Caulobacter crescentus*. *Nucleic Acids Research* **35**, e137 (2007).
7. Bendezu F. O., Hale C. A., Bernhardt T. G. & de Boer P. A. RodZ (YfgA) is required for proper assembly of the MreB actin cytoskeleton and cell shape in *E. coli*. *EMBO Journal* **28**, 193-204 (2009).
