# Supplementary material for: An outer membrane porin-lipoprotein complex modulates elongasome movement to establish cell curvature in Rhodospirillum rubrum
Source: Nat Commun. 2024 Sep 2;15:7616. doi: 10.1038/s41467-024-51790-z (PMC11369160; doi:10.1038/s41467-024-51790-z)
Supplement: Supplementary file 3 — Description of Additional Supplementary Files [file 41467_2024_51790_MOESM3_ESM.pdf]

Description of Additional Supplementary Files:

Supplementary Data 1: Whole-cell proteome analysis of *R. rubrum* wild-type cells. The spreadsheets provide an iBAQ analysis of the whole-cell proteomics data obtained for *R. rubrum* wild-type strain S1 (n=4 independent samples) as well as a summary of the parameters used for the MaxQuant analysis.

Supplementary Data 2: Conservation of PapS. The spreadsheet lists the bacterial orders that include species with *bona fide* PapS homologs and the number of PapS homologs in representative members of the Rhodospirillales.

Supplementary Data 3: Single-particle tracking statistics. The spreadsheet gives an overview of the conditions used for the different single-particle tracking experiments and a summary of the diffusion parameters obtained from the indicated types of analyses.

Supplementary Data 4: Colocalization analysis. The spreadsheet gives a summary of the parameters used and the results obtained in colocalization studies of the indicated *R. rubrum* strains.

Supplementary Data 5: Mass spectrometric identification of proteins in detergent extracts of *R. rubrum* wild-type cells. The spreadsheet shows the gel pieces analysed and the relative abundance of the proteins identified in each of the samples.

Supplementary Data 6: Muropeptide analysis. The spreadsheet shows the abundance of different muropeptide species in *R. rubrum* wild-type (S1) and Δ*papS* cells (n=2 independent experiments per strain).

Supplementary Data 7: AlphaFold2 and AlphaFold-Multimer models. The zip archive contains the structural coordinates (pdb format) and the error estimates (json format) for the structural models used in this study.

Supplementary Movie 1: 3D-SIM reconstruction of PapS-mNG ribbons. Exponentially growing *R. rubrum* cells producing PapS-mNG (SP02) were elongated by cefalexin (Cfx) treatment and imaged on minimal-medium nutrient pads using threedimensional structured illumination microscopy (3D-SIM). The 3D structure was reconstructed using the 3D SIM2 module of the Zen Black software. Axis intervals: 1 µm.

Supplementary Movie 2: Time-lapse analysis of PapS ribbon formation. Exponentially growing *R. rubrum* cells producing PapS-mNG (SP02) were transferred onto nutrient pads (1% agarose) and imaged in 10 min intervals. Bar: 5 µm.

Supplementary Movie 3: FRAP analysis of PapS-mCh structures. Recovery of the fluorescence signal in an *R. rubrum* cell producing PapS-mCh (JR56) in a representative FRAP experiment. Exponentially growing cells were transferred onto nutrient pads (1% agarose), the fluorescence signal was partially bleached using a 561 nm solid-state laser (t=0 min), and fluorescence recovery was monitored in 10 sec intervals. Bar: 3 µm.

Supplementary Movie 4: Disruption of PapS ribbons upon gentle cell lysis. Exponentially growing *R. rubrum* cells producing PapS-mNG (SP02) were transferred onto nutrient pads containing lytic agents. Shown are time-lapse series of three representative cells (I-III). Bar: 3 µm.

Supplementary Movie 5: 3D-SIM reconstruction of PapS-mNG. Exponentially growing *R. rubrum* cells ectopically expressing *papS-mNG* from a low-copy number replicative plasmid (SP136) were elongated by cefalexin (Cfx) treatment and imaged on minimal-medium nutrient pads using three-dimensional structured illumination microscopy (3D-SIM). The 3D structure was reconstructed using the 3D SIM2 module of the Zen Black software. Axis intervals: 1 µm.

Supplementary Movie 6: 3D-SIM reconstruction of an outer-membrane binding-deficient PapS-mNG fusion protein. Exponentially growing *R. rubrum* cells producing a PapS-mNG variant lacking the outermembrane lipid anchor from a low-copy number replicative plasmid (SP137) were elongated by cefalexin (Cfx) treatment. Subsequently, they were imaged on minimal-medium nutrient pads using three-dimensional structured illumination microscopy (3D-SIM), and the 3D structure was reconstructed using the 3D SIM2 module of the Zen Black software. Axis intervals: 1 µm.

Supplementary Movie 7: 3D-SIM reconstruction of a peptidoglycan-binding deficient PapS-mNG fusion protein. Exponentially growing *R. rubrum* cells producing a PapS-mNG variant defective in peptidoglycan binding from a low-copy number replicative plasmid (SP145) were elongated by cefalexin (Cfx) treatment. Subsequently, they were imaged on minimal-medium nutrient pads using three-dimensional structured illumination microscopy (3D-SIM), and the 3D structure was reconstructed using the 3D SIM2 module of the Zen Black software. Axis intervals: 1 µm.

Supplementary Movie 8: 3D-SIM reconstruction of PapS-mNG and mCh-Por39 produced in the same cell. Exponentially growing *R. rubrum* cells producing PapS-mNG and mCh-Por39 (SP06) were elongated by cefalexin (Cfx) treatment and imaged on minimal-medium nutrient pads using three-

dimensional structured illumination microscopy (3D-SIM). The 3D structure was reconstructed using the 3D SIM2 module of the Zen Black software. Axis intervals: 1 µm.

Supplementary Movie 9: 3D-SIM reconstruction of mCh-Por39 in the Δ*papS* background. Exponentially growing *R. rubrum* cells producing mCh-Por39 in the Δ*papS* background (SP12) were elongated by cefalexin (Cfx) treatment and imaged on minimal-medium nutrient pads using three-dimensional structured illumination microscopy (3D-SIM). The 3D structure was reconstructed using the 3D SIM2 module of the Zen Black software. Axis intervals: 1 µm.

Supplementary Movie 10: 3D-SIM reconstruction of PapS-mNG and mCh-Por39$_{D71S}$ produced in the same cell. Exponentially growing *R. rubrum* cells producing PapS-mNG and mCh-Por39$_{D71S}$ (SP130) were elongated by cefalexin (Cfx) treatment and imaged on minimal medium nutrient pads using three-dimensional structured illumination microscopy (3D-SIM). The 3D structure was reconstructed using the 3D SIM2 module of the Zen Black software. Axis intervals: 1 µm.

Supplementary Movie 11: 3D-SIM reconstruction of PapS-mNG and mChPor39 produced in the *por41*$_{D71S}$ background. Exponentially growing *R. rubrum* cells producing PapS-mNG and mCh-Por39 in the *por41*$_{D71S}$ background (SP131) were elongated by cefalexin (Cfx) treatment and imaged on minimal-medium nutrient pads using three-dimensional structured illumination microscopy (3D-SIM). The 3D structure was reconstructed using the 3D SIM2 module of the Zen Black software. Axis intervals: 1 µm.

Supplementary Movie 12: 3D-SIM reconstruction of porin-binding-deficient PapS-mNG and mCh-Por39 produced in the same cell. Exponentially growing *R. rubrum* cells producing PapS$_{W22A/W58A}$-mNG and mCh-Por39 (SP146) were elongated by cefalexin (Cfx) treatment and imaged on minimal-medium nutrient pads using three-dimensional structured illumination microscopy (3D-SIM). The 3D structure was reconstructed using the 3D SIM2 module of the Zen Black software. Bar intervals: 1 µm.

Supplementary Movie 13: 3D-SIM reconstruction of a porin-binding-deficient PapS-mNG fusion protein. Exponentially growing *R. rubrum* cells producing a PapS-mNG variant defective in porin binding from a low-copy number replicative plasmid (SP197) were elongated by cefalexin (Cfx) treatment and imaged on minimal-medium nutrient pads using three-dimensional structured illumination microscopy (3D-SIM). The 3D structure was reconstructed using the 3D SIM2 module of the Zen Black software. Axis intervals: 1 µm.

Supplementary Movie 14: 3D-SIM reconstruction of a peptidoglycan- and porin-binding-deficient PapS-mNG fusion protein. Exponentially growing *R. rubrum* cells producing a PapS-mNG variant defective in peptidoglycan and porin binding from a low-copy number replicative plasmid (SP174)

were elongated by cefalexin (Cfx) treatment and imaged on minimal-medium nutrient pads using three-dimensional structured illumination microscopy (3D-SIM). The 3D structure was reconstructed using the 3D SIM2 module of the Zen Black software. Axis intervals: 1 µm.

Supplementary Movie 15: 3D-SIM reconstruction of PapS-mCh and mNG-RodZ produced in the same cell. Exponentially growing *R. rubrum* cells producing PapS-mCh and mNG-RodZ (SP163) were elongated by cefalexin (Cfx) treatment and imaged on minimal-medium nutrient pads using three-dimensional structured illumination microscopy (3D-SIM). The 3D structure was reconstructed using the 3D SIM2 module of the Zen Black software. Axis intervals: 1 µm.

Supplementary Movie 16: Short-term timelapse series showing mNG-RodZ in the wild-type background. Exponentially growing *R. rubrum* cells producing mNG-RodZ (SP160) were transferred onto nutrient pads (1% agarose) and imaged in 1-sec intervals. Shown is the mNG-RodZ signal in a representative cell. Bar: 5 µm.

Supplementary Movie 17: Long-term timelapse series showing mNG-RodZ in the wild-type background. Exponentially growing *R. rubrum* cells producing mNGRodZ (SP160) were transferred o nto nutrient pads (1% agarose) and imaged in 2 min intervals. Bar: 5 µm.

Supplementary Movie 18: Short-term timelapse series showing mNG-RodZ in the Δ*papS* background. Exponentially growing *R. rubrum* cells producing mNG-RodZ in the Δ*papS* background (SP162) were transferred onto nutrient pads (1% agarose) and imaged in 1-sec intervals. Shown is the mNG-RodZ signal in a representative cell. Bar: 5 µm.

Supplementary Movie 19: Long-term timelapse series showing mNG-RodZ in the Δ*papS* background. Exponentially growing *R. rubrum* cells producing mNG-RodZ in the Δ*papS* background (SP162) were transferred onto nutrient pads (1% agarose) and imaged in 2-min intervals. Bar: 5 µm.
